# Mutational bias and the protein code shape the evolution of splicing enhancers

**DOI:** 10.1038/s41467-020-16673-z

**Published:** 2020-06-05

**Authors:** Stephen Rong, Luke Buerer, Christy L. Rhine, Jing Wang, Kamil J. Cygan, William G. Fairbrother

**Affiliations:** 1 Center for Computational Molecular Biology, Brown University, Providence, RI 02912 USA; 2 Ecology and Evolutionary Biology, Brown University, Providence, RI 02912 USA; 3 Molecular Biology, Cell Biology and Biochemistry, Brown University, Providence, RI 02912 USA; 4 Hassenfeld Child Health Innovation Institute of Brown University, Providence, RI 02912 USA

**Keywords:** Evolutionary genetics, Molecular evolution, RNA splicing

## Abstract

Exonic splicing enhancers (ESEs) are enriched in exons relative to introns and bind splicing activators. This study considers a fundamental question of co-evolution: How did ESE motifs become enriched in exons prior to the evolution of ESE recognition? We hypothesize that the high exon to intron motif ratios necessary for ESE function were created by mutational bias coupled with purifying selection on the protein code. These two forces retain certain coding motifs in exons while passively depleting them from introns. Through the use of simulations, genomic analyses, and high throughput splicing assays, we confirm the key predictions of this hypothesis, including an overlap between protein and splicing information in ESEs. We discuss the implications of mutational bias as an evolutionary driver in other cis-regulatory systems.

## Abstract (web summary)

Splicing is regulated by cis-acting elements in pre-mRNAs such as exonic or intronic splicing enhancers and silencers. Here the authors show that exonic splicing enhancers are enriched in exons compared to introns due to mutational bias coupled with purifying selection on the protein code.

## Introduction

Splicing refers to the process of removing long intervening sequences (i.e. introns) from pre-mRNAs and is catalyzed by the spliceosome, a large dynamic macromolecule that rivals the ribosome in complexity. Living cells are comprised of many such complex networks of interacting parts. These interactions arise from two macromolecular surfaces encoded by separate loci that co-evolved to specifically recognize and bind each other. They can be protein-protein contacts. They can also be *trans*-acting factors that bind a nucleic acid recognition element. Prior studies of the evolution of *cis*-regulatory networks often focused on later events such as the evolution of new specificity in existing *trans*-acting factors^1–8^. The evolutionary reconstruction of large families of similar transcription or splicing factors suggests that gene duplication followed by neofunctionalization was a primary mechanism for the recent expansion of *trans*-acting factor/*cis*-element networks in higher eukaryotes^1,6,8–12^. In contrast to later events, there is less insight into how initial recognition partnerships between *trans*-acting factors and *cis*-elements can form^1,3,7^. For example, how can a category of *cis*-elements acquire the correct ensemble of locations in the genome prior to the evolution of the recognition event?

This study investigates how exonic splicing enhancers (ESE) became enriched in exons relative to introns prior to their recognition by splicing activators. ESEs are short motifs that are located in exons and are necessary for pre-mRNA splicing^13^. A key property of ESEs is their enrichment in exons relative to introns^14^. ESEs are often recognized by SR proteins, a family of splicing factors that typically act as activators when bound in exonic sequence and repressors when bound in the intron^15^. ESEs also occur infrequently in introns. It has been demonstrated that ESE motifs disrupt splicing when relocated to an intron^16–18^. This result suggests that intronic occurrences of ESE motifs should be subject to purifying selection^14^.

The sequences that can function as exonic splicing enhancers (ESE) have been identified through a variety of methods. Some approaches are purely computational and infer *k-*mer function based on distribution around splice sites^14,19–21^, whereas other ESE models are determined empirically^22–26^. A recent empirical approach was used to assign an Enrichment Index (EI) score to all possible hexamers based on their ability to enhance splicing from several exonic positions in multiple minigene substrates^24^.

Similar minigenes have been used to screen mutations for their ability to cause splicing defects^27–30^. Although most disease-causing exonic mutations are presumed to affect the protein code, approximately one in three disease-causing mutations also affect splicing^21^. Mutations do not occur uniformly in a sequence but are strongly influenced by sequence context^31–34^. Moreover, mutational biases have evolved over time^35–38^. In many vertebrates, CpG nucleotides are highly mutable because of cytosine methylation, where a spontaneous deamination of methylated cytosine results in a C to T transition during replication^36^. The CpG motif has been associated with mutational hotspots for disease^39–41^ and stronger purifying selection^42^. Because of loss to mutation, CpG dimers are depleted in all regions of the human genome, but are more abundant in exons because many of the remaining exonic CpGs are evolving under strong purifying selection^42^. CpGs are one example of context dependent mutation rates. More sophisticated models leveraging large variant datasets have recently been used to estimate the relative mutation rate of nucleotides based on different *k*-mer sequence contexts^32,33^.

In this study, we test the hypothesis that mutational bias in conjunction with purifying selection on the protein code created precursor ESEs (pre-ESEs), motifs that were enriched in exons relative to introns which later evolved into ESEs. As the immediate sequence context can strongly influence the probability of a mutation, we reasoned that hypermutable motifs are rapidly depleted from the genome. However, hypermutable motifs that encode important protein motifs should be retained in exons by purifying selection. Since ESEs need to be enriched in exons relative to introns, we hypothesize this passive mechanism created pre-ESEs with an ESE-like distribution to which RNA binding proteins could later adapt. If this hypothesis is correct, ESEs would be (a) hypermutable (to facilitate their depletion from introns) and (b) retained by purifying selection on the protein code. Here we demonstrate mutable sequence motifs are evolving under higher levels of purifying selection. We utilize high-throughput biochemical assays, genomic analyses, and simulations to show that ESEs are highly mutable sequences that have been retained in exons because of selection on their protein-coding function. The passive role of background mutational processes in shaping *cis*-regulatory networks is also explored for other types of recognition elements.

## Results

### Mutational bias and protein-based selection create pre-ESEs

**Fig. 1:**
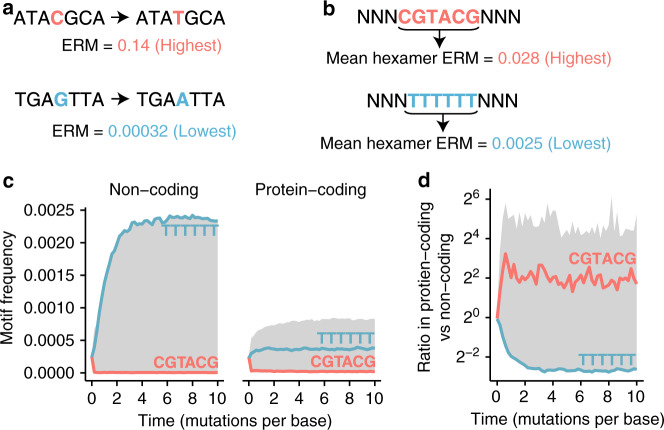
Mutational bias and protein-level selection shape motif evolution. **a** The highest (red)/lowest (blue) estimated relative mutation (ERM) rates based on 7-mer contexts as reported by Carlson et al.^32^. **b** The most (red)/least (blue) mutable hexamers based on the mean ERM rates over all possible single nucleotide changes and flanking sequence contexts. **c** Simulations initialized on a genome of random sequence were used to model the effects of protein-coding constraint and mutability on the evolution of motif frequencies (*y* axis) over time (*x* axis, mutations introduced per base of sequence). The simulation was run under the following conditions: with the constraint of preserving amino acid identity (right panel) or without constraint (left panel). Motif frequencies are shown for the most (red)/least (blue) mutable hexamers. Gray band shows the range across all 4096 hexamers as it evolves over time. **d** The frequency ratio of motifs in simulations with versus without coding constraint (analogous to exon versus intron enrichment) rapidly increased/decreased for the most (red)/least (blue) mutable hexamers.

Initially, simulations were used to test the hypothesis that mutational bias in conjunction with purifying selection on the protein code could create motifs that were precursors to ESEs (pre-ESEs). These pre-ESE motifs would be short *k*-mers that are enriched in exons relative to introns. It has recently been shown that mutation rates can vary >400-fold across different sequence contexts^32^ (Fig. 1a). A program was written to simulate realistic substitution probabilities in a genome of random sequence over many generations (Methods). This synthetic genome was allowed to evolve without selection (i.e. genetic drift) or with negative selection that disallows any change to the protein sequence (i.e. strict purifying selection). Substitutions were drawn in proportion to recently published estimated relative mutation (ERM) rates based on heptamer contexts^32^. Mutational bias has an enormous impact on sequence composition during simulated evolution. Sequence motifs, like CGTACG (Fig. 1b), associated with a high mutation rate are rapidly depleted (i.e. mutated to sequences associated with a lower mutation rate) (Fig. 1c, Non-coding, red lines), whereas sequences with low mutability, like TTTTTT (Fig. 1b), tend to accumulate in the synthetic genome (Fig. 1c, Non-coding, blue lines). Applying purifying selection by disallowing non-synonymous mutations reduces the magnitude but not direction of these changes (Fig. 1c, Protein-coding) such that the most mutable *k*-mer becomes ~3-fold enriched in regions under protein selection (i.e. regions analogous to coding exons) and the least mutable sequence becomes ~6-fold underrepresented in regions under protein selection (Fig. 1d). This experiment demonstrates how mutational bias in conjunction with selection on the protein code can generate *k*-mers that have an ESE-like distribution, enriched in exons relative to introns.

**Fig. 2:**
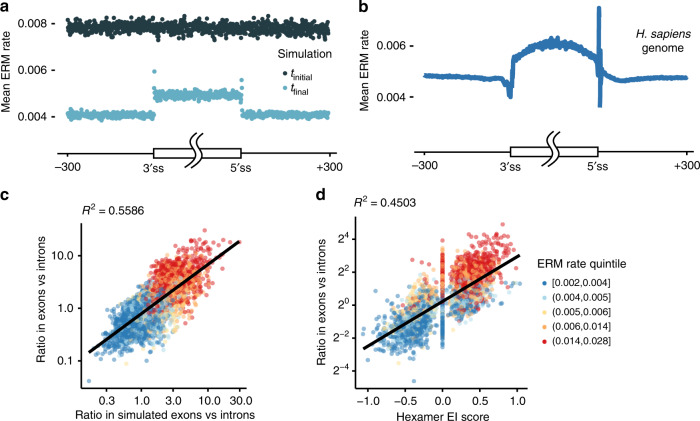
Protein-coding regions exhibit higher mutability than non-coding regions. **a** Simulations initialized on a genome of random sequence (*t*_initial_) were run to model evolution with or without the constraint of maintaining protein identity (*t*_final_). Mean ERM rate (*y* axis) in aggregate simulation data with (exonic) or without (intronic) constraint was plotted as a function of position (Methods). **b** Mean ERM rate (*y* axis) in aggregate human exon and intron data was plotted as a function of distance from annotated 3′ and 5′ splice sites. **c** Hexamers enriched in protein-coding versus non-coding simulations are positively correlated with hexamers enriched in human exons versus introns (Pearson’s correlation test, *p* < 2.2 × 10^−^^16^, *n* = 4096 hexamers), and exhibit higher mutability (greater ERM rate quintiles). **d** Mutable motifs are enriched in exons versus introns and exhibit high ESE activity: hexamers with higher exonic spicing enhancer activity, as measured by EI scores (*x* axis), are positively correlated with higher enrichment in human exons versus introns (*y* axis) (Pearson’s correlation test, *p* < 2.2 × 10^−^^16^, *n* = 4096 hexamers), and exhibit higher mutability (greater ERM rate quintiles). Source data are provided as a Source Data file.

To explore evidence of this phenomenon in human exons, several comparisons were undertaken. The simulation demonstrates highly mutable sequences are preferentially lost to mutation (Fig. 1c). However, the constraint of the protein code prevents the loss of some exonic sequences, leaving exons with a higher average mutation rate than introns (Fig. 2a). To test whether human exons have an elevated level of mutation relative to introns, the average mutation rate (for all three possible substitutions) at each position in human exons and introns was calculated and plotted as a function of distance from splice sites (Fig. 2b). The estimation of mean mutation rates in human exons and their flanking introns suggests human protein-coding sequence is ~25% more mutable than non-functional introns (Fig. 2b). This finding was independently validated by an analysis of a large exome variant dataset^43^. Rare variants occurred at sites with a higher average mutability than common variants, suggesting stronger purifying selection is acting in more mutable regions (Supplementary Fig. 1). This result is also consistent with prior observations of higher CpG levels in exons^44^, conservation of CpG levels at matched codon positions^42^, higher de novo mutation rates in exons^45^, and positive correlation between site-level conservation and de novo mutation rates in exons^41^. Taken together, the genomic and population genetic analyses corroborate the simulation’s finding that certain mutable sequences were preserved in exons by purifying selection on their protein-coding function. Indeed, the high degree of correlation between *k*-mer composition in exons and the simulation indicates the same hexamers enriched in human exons are preserved during the simulated evolution of constrained protein sequence (Fig. 2c, *R*^2^ = 0.56 positive slope). This correlation suggests simple models of mutational bias and purifying selection can account for more than half of the variability in exonic to intronic *k*-mer enrichment. The subset of *k*-mers that are enriched in exons have the correct distributional profile to be ESEs, but had not been shown to satisfy the second requirement of ESE function, the ability to bind splicing activators.

### The most mutable sequences in human exons are ESEs

To determine whether these highly mutable sequences enriched in exons can bind any of the numerous splicing activators present in cells, each hexamer was associated with an empirically determined enhancer activity score, the EI^24^. A hexamer’s EI score was found to correlate reasonably well with a hexamer’s enrichment in human exons relative to introns (Fig. 2d, *R*^2^ = 0.45 positive slope). An ESE would be expected to be enriched in exons relative to introns and have a high EI score (Fig. 2d, upper right quadrant). The ERM rate of possible mutations was associated with each hexamer. The subset of hexamers predicted to possess ESE activity coincide with the hypermutable regions of the genome (Fig. 2d, red dots in upper right quadrant), suggesting that human ESEs are made of the most mutable sequences. The same effect was observed to the same (or greater) extent with two other ESE models^25^ (Supplementary Fig. 2). This phenomenon is not strictly a function of CpG-containing *k*-mers as repeating the analysis without CpG resulted in the same trend (solid line) in all studies (Supplementary Fig. 3a–c). Furthermore, the high correlation between exon enrichment in vivo and in silico (Fig. 2c) suggests that the ESE motifs observed in human exons arose spontaneously through the background mutation process in exons evolving under purifying selection. This result demonstrates ESEs are highly mutable and ESE-like distributions will form passively without requiring selective forces related to their eventual function in splicing.

### Selection against stop gain mutations preserve ESEs

**Fig. 3:**
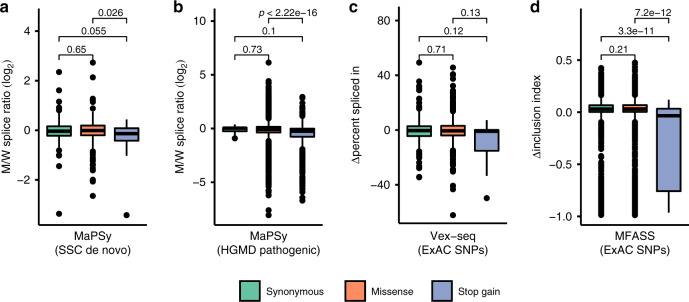
Stop gain mutations are the most disruptive to splicing function. Relationship between a mutation’s effect on splicing (*y* axis) was plotted for progressively more disruptive categories of protein disruption (synonymous < missense < stop gain) based on data from four high-throughput splicing assays. **a** Boxplot of MaPSy M/W splice ratios for a panel of de novo mutations from the Simons Simplex Collection (SSC) (*n* = 192 synonymous, 413 missense, 31 stop gain variants). M/W splice ratios were derived from mutant and wild-type read counts in a high-throughput splicing assay, based on summed read counts over two input sequencing and four output transfection replicates. **b** Boxplot of MaPSy M/W splice ratios for previously studied pathogenic variants from the Human Gene Mutation Database (HGMD) (*n* = 8 synonymous, 2877 missense, 822 stop gain variants). **c**, **d** Boxplots for measures of splice disruption for single nucleotide polymorphisms (SNPs) from the Exome Aggregation Consortium (ExAC) previously studied using **c** Vex-seq (Δpercent spliced in, *n* = 244 synonymous, 520 missense, 18 stop gain variants) and **d** MFASS (Δinclusion index, *n* = 4416 synonymous, 8290 missense, 77 stop gain variants). **a**–**d** The boxplots indicate the median (middle line), first and third quartiles (box), and 1.5× IQR (whiskers); pairwise *p-*values from Mann–Whitney two*-*tailed test. Source data are provided as a Source Data file.

It was surprising that the correlation between hexamer composition in the simulation and human exons was strong. The stringent implementation of purifying selection on protein sequence (disallowing all non-synonymous mutations) is an obvious simplification of the selective forces under which proteins evolve. To understand the nature of the protein-based selection, a closer examination of mutations that disrupt splicing was undertaken. We reasoned that if ESEs were shaped by purifying selection on proteins, mutations that disrupt splicing should also be disruptive to the protein code. A dataset of naturally occurring de novo mutations was used because they capture endogenous mutational bias with a minimal role of selection in their ascertainment^46^. This dataset of 707 de novo mutations was engineered into a high-throughput splicing reporter assay and analyzed using the MaPSy protocol^29^ (Methods, Supplementary Data 1). Briefly, the wild-type and mutant version of the exon with intron flanks was incorporated into a splicing minigene reporter (Supplementary Fig. 4a, b). All 1414 reporters were pooled for transfection into HEK 293T tissue culture cells in four replicates (Supplementary Fig. 5). Sequencing was used to estimate the skew in mutant/wild-type allelic ratio (M/W splice ratio) in the starting pool relative to the successfully spliced fraction (Supplementary Fig. 4a). Within this set of 707 exonic mutations, 54 mutations fell within the exonic portion of the 3′ and 5′ splice site regions (i.e. the first and final 3 nucleotides of the exon). As expected, this class of variant was associated with the highest degree of disrupted splicing. Scoring both allelic versions for agreement to the splice site consensus indicated that a decrease in splicing occurs when the mutation reduces the match to the 5′ or 3′ss (Supplementary Fig. 4c). The remaining mutations comprised 192 synonymous, 413 missense, and 31 stop gain mutations. Stop gain mutations, which are the most disruptive to protein function, were also found to be the most disruptive to splicing (M/W splice ratios, Fig. 3a). Missense and synonymous mutations disrupted splicing to a lesser extent than stop gain mutations. To place these results in deeper context, different datasets of variants, each with different ascertainment (de novo mutations, disease alleles, and exome variants), were explored for their effect on splicing (Fig. 3b–d). As expected, disease-causing variants^29^ were more deleterious in the missense and stop gain categories (Fig. 3b). Two splicing studies utilizing exome variants were also reanalyzed^28,30^ (Fig. 3c, d). Here, missense and synonymous variants had little effect on splicing. However, a commonality across all four studies was a strong association between stop gain mutations and splicing disruption. This was true even when we considered MaPSy in vitro splicing assay results^29^, which should not be affected by nonsense-mediated decay (Supplementary Fig. 4d). The proposed hypothesis argues that protein-based purifying selection maintains mutable pre-ESE motifs in coding exons while depleting them from introns. These high-throughput biochemical experiments on thousands of naturally occurring variants suggests the selection that retains ESEs is largely negative selection against the creation of stop codons.

### Mutation’s effects on protein and splicing are correlated

**Fig. 4:**
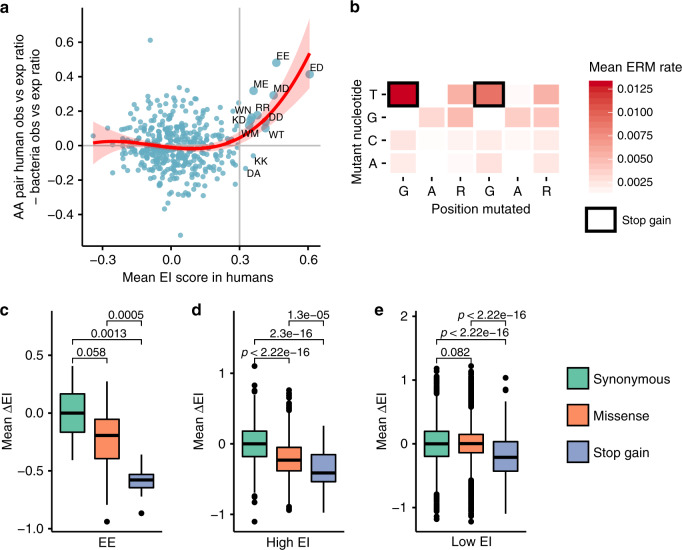
ESE activity is associated with specific amino acid pairs. **a** All 4096 hexamers were associated with exonic splicing enhancer activity (mean EI) and the amino acid (AA) doublet motifs they encode in genomes with splicing (human) or without splicing (bacteria). The enrichment of AA pairs in human relative to bacterial genomes was analyzed as a function of mean EI score. AA pairs with EI score >0.3 and human enrichment >1 are highlighted (upper right). Cubic polynomial regression (95% confidence band). **b** EE corresponds to the GARGAR ESE. The mutability of GARGAR mutations at each position was plotted (mean ERM rate based on an average over flanking contexts of GARGAR in the human genome). Stop gains have high mutability (black boxes). **c**–**e** All possible mutations in the human genome were binned by their predicted effect on protein function, scored for their change in ESE activity (mean ΔEI), and assigned to the amino acid pair in which they belong. Severity of splice disruption was found to be concordant with severity of protein disruption (synonymous < missense < stop gain) for **c** EE/GARGAR (*n* = 8 synonymous, 56 missense, 8 stop mutatio*n*s), **d** high EI (>0.3) (*n* = 306 synonymous, 980 missense, 64 stop gain mutations), **e** but not low (<0.3) EI AA pairs (*n* = 16,010 synonymous, 46,732 missense, 2742 stop gain mutations). **c**–**e** The boxplots indicate the median (middle line), first and third quartiles (box), and 1.5× IQR (whiskers); pairwise *p-*values from Mann–Whitney two-tailed test. Source data are provided as a Source Data file.

One consequence of protein selection as a driver of ESE evolution is that certain protein motifs should be associated with splicing^23^. To determine whether such motifs exist, each exonic occurrence of a hexamer was mapped and translated in the appropriate reading frame. The two amino acids with the greatest overlap with the hexamer was associated with the hexamer’s EI score. An average EI score for all 400 possible amino acid pairs was tabulated from the weighted ensemble of hexamer EI scores that encoded the amino acid pair in the human genome. Some amino acid pairs are overrepresented in the proteome (i.e. occur more frequently than the product of their single amino acid frequencies). It is possible that these amino acid pairs are enriched because of the need to encode ESEs. It is also possible these amino acid pair enrichments reflect nearest-neighbor correlations driven by requirements of protein structure. We reasoned that protein-based patterns would also occur in prokaryotic proteomes, whereas enrichments driven by splicing would be restricted to species that had introns. To make this distinction, an amino acid pair’s EI score was compared to its enrichment in the human proteome and also an aggregate of non-splicing proteomes (Methods). Amino acid pairs associated with high ESE activity are enriched in the human proteome but not in non-splicing bacterial proteomes (Fig. 4a). The greatest observed difference in enrichment in amino acid pairs are consecutive occurrences of glutamic acid, EE (Fig. 4a). This amino acid pair corresponds to the classic GARGAR ESE motif^14^.

EE is by far the most abundant amino acid ESE motif and the second most abundant amino acid pair in the human proteome. Simulating the mutation process using human exons and mutational bias (Methods) found this EE motif to be among the most likely of all 400 amino acid pairs to mutate to a stop codon (~1/3 of mutations created stops; Supplementary Fig. 6), suggesting purifying selection against stop gain mutations played a major role in the evolution of the GARGAR ESE. As only 1/9 of all possible substitutions in GARGAR can create in-frame stop codons, and transversions typically occur with lower probability than transitions, it seemed unlikely that a third of all mutations in this ESE should result in stop codons. However, the two G>T mutations in this purine-rich context represent an unreported hotspot motif occurring at twice the frequency of transitions and approximately half the frequency of CpG (Fig. 4b). The initial hypothesis proposed that context dependent elevated mutation rate (i.e. mutational bias) coupled with protein selection drives certain *k*-mers to adopt an ESE-like distribution (i.e. enriched in exons, depleted in introns). The classic GARGAR ESE appears to satisfy both criteria in that the highest mutation rates create the greatest disruption to the encoded protein. Considering the spectrum of mutations that can occur in GARGAR, there is a strong agreement between the deleteriousness of the predicted protein effect of the mutation and the predicted splicing effect (Fig. 4c; change in EI for synonymous < missense < stop gain). Although this relationship was discovered for GARGAR, nearly all ESE protein motifs exhibit the same correlation between rank order of variant deleteriousness to protein and splicing function (Fig. 4d, e). These results, together with the observation that amino acid pairs that encode ESEs are enriched in splicing genomes (Fig. 4a), suggest a strong signature of protein-based selection on the evolution of ESEs.

### Mutational bias and the protein code drive evolution of ISEs

**Fig. 5:**
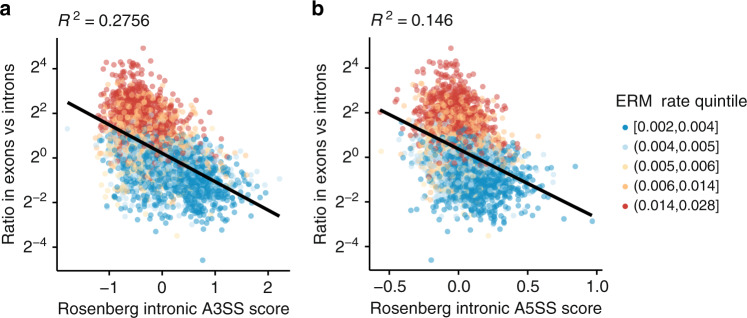
Mutational bias and selection on the protein code shape the evolution of ISEs. Scores of intronic splicing enhancer (ISE) activity^25^ (*x* axes) are negatively correlated with motif-level mutability (ERM rate quintiles) and exonic versus intronic enrichment in the human genome (*y* axis). This is the opposite of the positive correlation seen in Fig. 2c, d and Supplementary Fig. 2a, b for ESEs. Rosenberg **a** intronic A3SS and **b** intronic A5SS scores of ISE activity are based on the effect (log_2_ odds ratio) of intronic hexamers on alternative 3′/5′ splice site usage as measured by high-throughput minigene experiments. **a**, **b** Squared Pearson’s correlations (*n* = 4096 hexamers). Source data are provided as a Source Data file.

The observation that selection on the protein code coupled with mutational bias can create pre-ESEs raises questions about other types of gene expression signals. For example, intronic splicing enhancers (ISE) are enriched in introns relative to exons. Our initial exploration of the effects of mutation rate on intron/exon sequence composition suggests sequences of low mutability tend to accumulate in introns (Fig. 1c, blue line). Reimplementing the analysis of Fig. 2d confirms intronically enriched motifs that possess predicted ISE activity^25^ have a low level of mutability (Fig. 5 and Supplementary Fig. 3d, e). This offers further examples of how selection against variants that alter the protein code can shape the evolution of non-coding signals.

### A model for ESE and ISE evolution

**Fig. 6:**
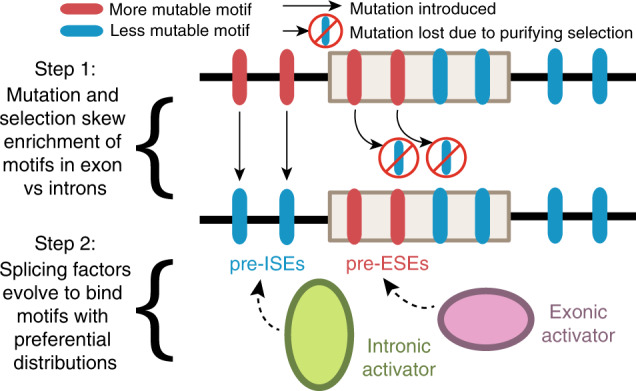
A two-step model for the evolutionary origins of ESEs and ISEs. (Step 1) Genomes consist of a mixture of more mutable (red) and less mutable (blue) motifs. More mutable motifs are removed from the genome over time and replaced by less mutable motifs (arrows from red to blue). In coding exons, more mutable motifs may instead be retained by purifying selection acting to preserve the protein sequence (blocked arrows from red to blue motifs). Thus, mutation and selection jointly drive a skewed enrichment of motifs in exons relative to introns. More mutable motifs become enriched in exons (pre-ESEs) and less mutable motifs become enriched in introns (pre-ISEs). (Step 2) The initial evolution of binding specificity between splicing factors and *cis*-elements should be predicated on the background motif distributions shaped by mutational bias and protein-level selection. Exonic splicing activators (pink) will tend to evolve to bind pre-ESEs, which are already enriched in exons. Intronic splicing activators (green) will tend to evolve to bind pre-ISEs, which are already enriched in introns.

In summary, we present the hypothesis that mutational bias in conjunction with selection on the protein code gave rise to pre-ESE (and pre-ISE) motifs with exonic (and intronic) distributions to which splicing activators later adapted their binding specificity (Fig. 6). A mathematical model of motifs evolving with varying degrees of mutational bias and selection (Supplementary Note 1) demonstrates that both forces are required to generate pre-ESE (and pre-ISE) distributions (Supplementary Fig. 8). As mutational bias increases, motif enrichment in introns becomes more variable (Supplementary Fig. 8a). However, as strength of purifying selection increases, motif evolution in exons becomes more constrained and less variable (Supplementary Fig. 8b), resulting in greater differences in motif enrichment in exons versus introns (Supplementary Fig. 8c). The main ESE, the GARGAR motif, illustrates both of these properties (i.e. strong selection against and high mutation rate for stop gains). It is interesting to consider the utility of high mutability in reducing background occurrences of a signal sequence, and perhaps consider other major signals, such as CpG islands in vertebrate promoters, as additional examples of this phenomenon. It is somewhat counterintuitive to regard certain classes of functional elements as intrinsically more fragile than other sequences. There is great interest for clinical genetics in predicting the splicing effects of variants outside the canonical splice sites. This work suggests exonic splicing mutations may occur more frequently than intronic splicing mutations in clinical genetics.

## Discussion

We present a parsimonious model for the evolution of exonic splicing enhancers (ESEs). Counter to intuition, purifying selection leaves functional elements more mutable than non-functional regions of the genome. Constraint imposed by purifying selection on the protein code creates a favorable distribution of mutable *k-*mers in exons relative to introns through the background mutational process (Fig. 6). Mutator phenotypes can alter mutational bias, and there is evidence for evolution in mutational spectra across human populations, mammal species, and eukaryotic lineages^35–38^. It is thus unlikely that sequence composition ever reaches a mutation-selection equilibrium. Moreover, the stringency of selection varies across and even within proteins. Although the effects of these uncertainties can be explored by varying simulation parameters (Supplementary Fig. 9) and mathematical modeling (Supplementary Fig. 8), our simulations demonstrate how this passive mechanism with mutational bias and constraint of the protein code can create pre-ESE *k-*mers with all the properties of an ESE (i.e. high EI scores and high exon/intron occurrence frequency). Similar processes can create pre-ISEs in introns (Fig. 5).

It has been suggested that exons can accommodate interweaved protein and splicing information by exploiting non-overlapping coding and non-coding sites, respectively, due to the redundancy in the protein code^47–51^. Our model instead predicts a large proportion of the information coding for ESEs must be coincident with the information coding for proteins (simulations suggests ~50%, Supplementary Fig. 9d). Many mutations that disrupt protein function have also been shown to disrupt splicing function^29^. High-throughput splicing analysis indicated selection against stop gain mutations is a primary driver of ESE evolution, but a signature of protein-based selection was not seen for missense mutations (Fig. 3). One explanation for this loss of resolution is that splicing assays return both mutations that create silencers and mutations that disrupt enhancers. Analyzing mutation in enriched amino acid pairs is instead focused on disruption of positive signals (i.e. ESEs), and in this analysis it can be seen that missense mutations disrupt splicing more than synonymous variants (Fig. 4 and Supplementary Fig. 7). It has also recently been shown that exons co-regulated by the same splicing factors have similar nucleotide composition bias and code for physiochemically similar amino acids^52^. Together, these results support the predicted overlap between protein and splicing codes in ESEs.

After the establishment of a network of ESEs and their *trans*-acting splicing factors, it is certainly possible that the splicing code has broadened to include information at synonymous sites. Synonymous mutations have been shown to disrupt splicing and contribute to disease^28,30,53,54^, and studies of selective constraint^48,49,51^ and distributions of fitness effects^55^ support the functional role of synonymous sites in ESEs. In addition, ESEs, once established, will co-evolve with their *trans*-acting partners due to selection on the splicing phenotype^2,5^, and differences in genetic drift and genome size across eukaryotic lineages can affect the efficacy of mutation and selection processes^56^. Thus, there are many additional forces that shape the evolution of the splicing code. Nevertheless, mutational bias and protein selection should continue to act passively to maintain favorable ESE distributions, and changes to either can provide novel opportunities for ESE evolution.

For non-coding recognition elements, we propose mutational bias can play a profound, indirect role in their evolution. Although the co-evolution of an element and the binding specificity of an activator is important, functional specificity is impossible if the element occurs ubiquitously. The emergence of well-known recognition elements such as CpG islands and ESEs may have been driven by their mutability, which conferred a high signal-to-noise ratio by ensuring a low frequency of occurrence in the genomic background.

## Methods

### Estimated relative mutation (ERM) rates

Estimated relative mutation (ERM) rates for heptamers were downloaded from a previous study^32^. Each heptamer is associated with three ERM rates for each of the three single nucleotide changes at the middle position (e.g. AAA[A]AAA > AAA[T]AAA, AAA[C]AAA, AAA[G]AAA). Mean hexamer-level ERM rates were calculated as the average of heptamer-level ERM rates over all 18 possible single nucleotide changes in the hexamer (3 mutations for each position), and assuming uniform occurrence of all ±3 nt flanking sequence contexts (e.g. NNN[AAAAAA]NNN).

### Exonic and intronic splicing enhancer scores

Hexamer-level scores of ESE/ISE activity based on high-throughput minigene assays were downloaded from two previous studies. Enrichment index (EI) scores were downloaded from a previous study^24^. Rosenberg scores of ESE (exonic A3SS, exonic A5SS) and ISE (intronic A3SS, intronic A5SS) activity^25^ were recalculated by running Notebook 4: Estimating Motif Effects available at https://github.com/Alex-Rosenberg/cell-2015/tree/master/ipython.notebooks.

### Simulations with mutational bias and protein selection

Simulations were initialized on a genome of 5000 random DNA sequences each of length 999 nt. Each sequence was translated into their corresponding amino acid sequence for simulations with selection. Sequences were evolved one substitution at a time. In each time step, a position was mutated as follows: Step (1) each position was assigned the mean ERM rate of the three possible single nucleotide changes based on current heptamer context; Step (2) a position was selected to mutate based on its ERM rate relative to that of other positions; Step (3) a mutant allele was selected based on the ERM rates of the three possible single nucleotide changes; Step (4a) in simulations with protein-coding constraint, a mutant allele is introduced if it does not change the amino acid sequence and if it does not create a stop codon, otherwise the mutation is rejected; Step (4b) in simulations without constraint, the mutation is introduced. Sequences were circularized in silico to account for edge effects at positions ≤3 nt from the ends.

As the simulations progressed, the state of the simulation was recorded at time intervals of 0.2 mutations per base (mutations per base = the number of mutations introduced scaled by the total length of sequences in base pairs) up to 10 mutations per base (allowing plenty of time for the simulations to reach a dynamic mutation-selection equilibrium). The mean ERM rate of a sequence was calculated as the mean of all heptamer ERM rates for all possible single nucleotide changes. The mean Enrichment Index (EI) score^24^ was calculated as the mean of all hexamer EI scores. Individual hexamer frequencies were recorded to compute their enrichment in simulated exons versus introns. Mean ERM rates and mean EI scores were calculated over all sequences weighted by sequence length.

To vary mutational bias, the ERM rates used in Step 1 of the simulation were modified as follows: Step (1) the ERM rates were log-transformed; Step (2) the log-transformed ERM rates were multiplied by a mutational bias parameter (1: original mutational bias, 0: no mutational bias, 0.5: reduced mutational bias, and 2: increased mutational bias); Step (3) the rescaled log-transformed ERM rates were inverse log-transformed. This approach was used because: (a) it allows for the dynamic range of mutation rates (ERM rates) between the most and least mutable heptamers to be tuned with a single scaling parameter (the mutational bias parameter); (b) it preserves non-negativity and the rank order of ERM rates; and (c) it returns the original ERM rates when the mutational bias parameter is 1 and uniform ERM rates when it is 0. In Supplementary Fig. 9c, d, simulations were instead initialized on a genome of human exonic sequences based on UniProt SwissProt^57^ coding domain sequences for 21,328 protein-coding genes downloaded from the UCSC Table Browser^58^. UniProt SwissProt intronic sequences from the UCSC Table Browser were also used to compute genome-wide means ERM rates.

### ERM rates and EI scores of genomic regions

GENCODE V17 exon-intron coordinates and sequences were downloaded from the UCSC Table Browser^58^. Genomic windows 300 nt into the intron and 150 nt into the exon relative to each 3′ and 5′ splice site were annotated for their ERM rate. All positions were assigned an ERM rate based on the heptamer sequence spanning +3 to −3 nt of the given position. For exonic or intronic positions within 3 nt of an exon boundary, the heptamer was extended into the neighboring intron or exon. For a given position relative to the exon boundaries, ERM rates associated with that position were averaged genome-wide.

For simulated exons and introns, ~1/3 of the 5000 sequences were used as middle exons (using the simulations with protein-coding constraint), and ~2/3 of the remaining sequences were used as flanking introns (using the simulations without constraint). Each sequence was trimmed to the first 300 nt. Distinct triples of intron, exon, intron sequences were concatenated into 900 nt sequences (these are analogous to the concatenation of 3′ and 5′ genomic windows based on GENCODE exons). For a given pseudo-position from 1 to 900, ERM rates associated with that position were averaged across all concatenated sequences.

### Exome Aggregation Consortium (ExAC) analysis

ExAC variants were downloaded from a previous study along with derived allele frequency (DAF), ancestral/derived states, and predicted variant effects^43^. Variants with ambiguous ancestral state, or predicted variant effects other than intronic, synonymous, missense, or stop gain were removed. Extremely rare variants (DAF < 0.00005), whose site frequency spectrum is strongly affected by recurrent mutations at large sample sizes and high mutation rates^59^, were also removed. ERM rates were assigned to each site containing an ExAC variant based on the ancestral to derived nucleotide change at the site and the heptamer context based on human reference GRCh37/hg19. Mean ERM rates were calculated for all variants in each variant effect × DAF bin.

### De novo variant splicing assay

De novo variants from the Simons Simplex Collection^46^ were downloaded and mapped to the human reference GRCh37/hg19. 707 variants that mapped to exons ≤115 nt in length were selected for the splicing assay^29^ (Supplementary Fig. 4). A 180 nt window of endogenous sequence, which includes the exon of interest and either the wild-type or mutant allele, 15 nt of the downstream intron, and at least 50 nt of the upstream intron, was flanked by 25 nt primer sequences (forward primer: 5′-GTCCACCATACCTTCGATTGTCGCG-3′, reverse primer: 5′-ACCGTGCACCTACCGAATCTCCTTA-3′), yielding a 230-mer oligonucleotide library synthesized by Agilent Technologies.

The in vivo splicing minigene reporter construct includes a cytomegalovirus (CMV) promoter, exon 7 of ACTN4 exon with part of its downstream intron, the 230-mer de novo library, exon 10 of ACTN4 with part of intron 9 and the bGH poly(A) signal sequence (Supplementary Fig. 4b). Common sequences (everything except the 230-mer de novo library) were concatenated by overlapping PCR and cloned with TOPO TA (Invitrogen) to generate a 5′ common sequence and a 3′ common sequence. Equimolar amounts of the 5′ common sequences, the 3′ common sequences, and the oligonucleotide library (de novo 230-mers) were concatenated in a single PCR reaction. The PCR product was purified and size selected with Agencourt AMPure beads (Beckman Coulter). The in vivo minigene constructs were transfected into human embryonic kidney HEK 293T cells obtained from the American Type Culture Collection (ATCC# CRL-3216) in four cell culture replicates using Lipofectamine 3000 (Invitrogen) in a 6-well plate. HEK 293T is not listed in the ICLAC Register of Misidentified Cell Lines (v10), and was confirmed mycoplasma free in previous passage. Thirty hours after transfection, RNA was extracted using TRIzol (ThermoFisher) and treated with DNase (Invitrogen). cDNA was generated with SuperScript IV Reverse Transcriptase (Invitrogen) and random 9-mers. 20 cycles of PCR reactions (GoTaq, Promega) were carried out using the cDNA as template.

Input minigene reporters (in two technical replicates) and output spliced species for each of the four transfection were sequenced on an Illumina HiSeq 3000 (2 × 150). Reads from input and output sequencing were aligned to minigene reporter sequences using the STAR aligner^60^ (version 2.5.1b). For input alignment, split reads were not allowed, whereas for output alignment, split reads were allowed. Uniquely mapped reads with up to ten mismatches were tabulated for each input and output sequencing library, and were shown to be highly correlated across input and output replicates (Supplementary Fig. 5). The M/W splice ratio for each de novo variant was calculated as ${\mathrm{log}}_2\left( {\frac{{{\mathrm{mt}}_{\mathrm{o}}/{\mathrm{mt}}_{\mathrm{i}}}}{{{\mathrm{wt}}_{\mathrm{o}}/{\mathrm{wt}}_{\mathrm{i}}}}} \right),$ where mt_o_ is the count of mutant spliced species, mt_i_ is the count of mutant unspliced input, wt_o_ is the count of wild-type spliced species, and wt_i_ is the count of wild-type unspliced input, where we have used the sum of read counts across input and output replicates for each species (Supplementary Data 1).

### Annotations of splicing assay variants

MaPSy data for de novo mutations generated in this study were combined with previously published high-throughput splicing data from a study of disease-causing variants in HGMD^61^ (original MaPSy paper^29^), and two studies of ExAC exome variants^43^ (Vex-seq^30^ and MFASS^28^). All variants were annotated for their functional effect using SnpEff tool (version 4.3T) with default parameters and the -canon option^62^. 54 de novo variants annotated as splice region variants by SnpEff and were scored for change in 3′ or 5′ MaxEntScan splice site scores^63^. 17 variants annotated by SnpEff as structural interaction, 5′ UTR, 3′ UTR, and sequence feature variants were removed from downstream analysis. The remaining variants were binned by synonymous, missense, or stop gain effect, and compared to their measured splicing disruption in each assay’s respective units: log_2_ M/W splice ratio (MaPSy), Δpercent spliced in (Vex-seq), or Δinclusion index (MFASS).

### Analysis of nonsense-medicated decay (NMD)

MaPSy data for disease-causing mutations in HGMD in in vitro context (incubated in HeLa cell nuclear extract instead of transfected into HEK 293T cells) were downloaded from a previous study^29^, and annotated as synonymous, missense, or stop gain using SnpEff. In vitro splicing assay results for stop gain mutations should not be affected by NMD (which occurs in the cytoplasm), whereas NMD may be present in vivo (depending on if the minigene preserves the native reading frame of the exon).

### Associating EI scores with amino acid pairs

Using human exonic sequences from the Consensus Coding Sequence (CCDS) Project^64^, each occurrence of a hexamer was identified. The two codons with the largest overlap with the hexamer in the reading frame were translated into an amino acid pair and associated with that hexamer instance. The mean EI score for each amino acid pair was determined by taking a mean of the EI scores of the ensemble of associated hexamers weighted by the number of times the hexamer was translated to that particular amino acid pair.

### Amino acid pair enrichment in humans versus bacteria

Using human exonic sequences from the CCDS project^64^, a protein sequence set was generated by concatenating and translating exonic sequences for each gene. The frequency of each amino acid pair was determined in this protein sequence set and amino acid pair enrichment was calculated as the frequency of the amino acid pair divided by the product of the individual amino acid frequencies (observed/expected frequency ratio). This calculation was repeated using the set of protein sequences belonging to gammaproteobacteria from the Cluster of Orthologous Groups^65^.

### ΔEI of mutation types by amino acid pairs

Each amino acid pair can be coded by multiple codon pairs. Each codon pair can be mutated at each of six positions to each of three different nucleotides. For each of these possible mutations, we labeled the mutation as synonymous, missense, or stop gain; we computed a mean ERM rate for the mutation based on the heptamer contexts of all relevant positions in the human genome; and we computed a mean ΔEI score for the mutation as follows: each mutation was assigned the EI scores of the two hexamers where the mutation occurs in the third or fourth position, the difference between the mean EI score of the mutated and unmutated hexamers was assigned to that mutation, and finally the mean ΔEI was calculated over all relevant potential mutations in the human genome. We plot the distribution of mean ΔEI score for synonymous, missense, and stop gain mutation classes for individual amino acid pairs (Fig. 4c and Supplementary Fig. 7), and aggregates of amino acid pairs with high or low mean EI scores (Fig. 4d, e).

### Statistical methods

Statistical tests were performed using R software (version 3.6.1) for Pearson’s correlation tests and Mann–Whitney two-tailed tests where noted in figure legends.

### Reporting summary

Further information on research design is available in the Nature Research Reporting Summary linked to this article.

## Supplementary information

Supplementary Information

Reporting Summary

Description of Additional Supplementary Files

Supplementary Data 1

## Data Availability

The authors declare the data supporting the findings of this study are available within the paper and its supplementary files, and are available from the corresponding author upon reasonable request. The source data underlying Figs. 2c, d, 3a–d, 4a–e, 5a, b and Supplementary Figs. 1, 2a, b, 3a–e, 4c, d, 5a–g, 6, and 7 are provided as a Source Data File. Other datasets referenced in this study are available from the following web links: ERM rates [https://www.nature.com/articles/s41467-018-05936-5#Sec22], EI scores [https://genome.cshlp.org/content/21/8/1360/suppl/DC1], Rosenberg intronic and exonic A3SS and A5SS scores [https://github.com/Alex-Rosenberg/cell-2015/blob/master/ipython.notebooks/Cell2015_N4_Motif_Effect_Sizes.ipynb], ExAC variant annotations [https://github.com/macarthur-lab/exac_2015], MaxEntScan scores [http://hollywood.mit.edu/burgelab/maxent/Xmaxentscan_scoreseq.html], MaPSy in vivo and in vivo splicing assay results for HGMD pathogenic variants [http://fairbrother.biomed.brown.edu/data/bulk_download.txt], Vex-seq splicing assay results [https://github.com/scottiadamson/Vex-seq/blob/master/processed_files/delta_PSI_values.tsv], and MFASS splicing assay results [https://github.com/KosuriLab/MFASS/blob/master/processed_data/snv/snv_data_clean.txt]. Source data are provided with this paper.

## Source data

Source Data

## Code availability

All custom scripts for the evolutionary simulations and the mathematical model are available at https://github.com/stephenrong/mutation-paper (10.5281/zenodo.3727198, released under the MIT License). Source data are provided with this paper.
