# Supplementary material for: Mutational bias and the protein code shape the evolution of splicing enhancers
Source: Nat Commun. 2020 Jun 5;11:2845. doi: 10.1038/s41467-020-16673-z (PMC7275064; doi:10.1038/s41467-020-16673-z)
Supplement: Supplementary file 1 — Supplementary Information [file 41467_2020_16673_MOESM1_ESM.pdf]

Supplementary Information

Mutational bias and the protein code shape the evolution of splicing enhancers

Rong et al.

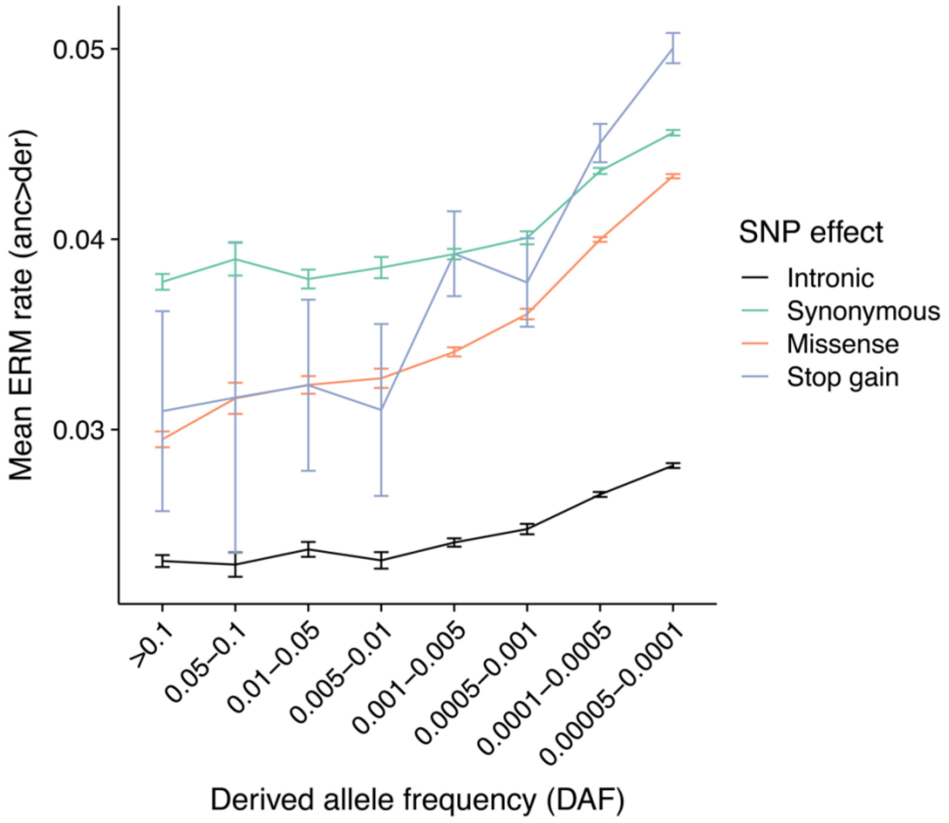

Supplementary Figure 1. Higher mutability at sites with stronger purifying selection. Exome Aggregation Consortium (ExAC) variants were binned by functional effect on protein (SNP effect) and derived allele frequency (DAF). Variants with DAF < 5e-5, whose allele count distributions are affected by recurrent mutations[59], were removed. The ancestral to derived change and 7-mer context was used to assign a mutability score (ERM rate) to each site with a variant. Mean ERM rate was calculated for variants in each bin (*n*=255,092 intronic, 244,412 synonymous, 382,930 missense, and 6,832 stop gain variants). Bars: mean ± s.e.m. In each functional category, rare variants were on average found at sites with higher mutability (mean ERM rate) than common variants, with greater slopes for variant categories that cause more severe protein disruption (stop gain > missense > synonymous & intronic). This indicates there is stronger selective/functional constraint acting at sites with higher mutability. Source data are provided as a Source Data file.

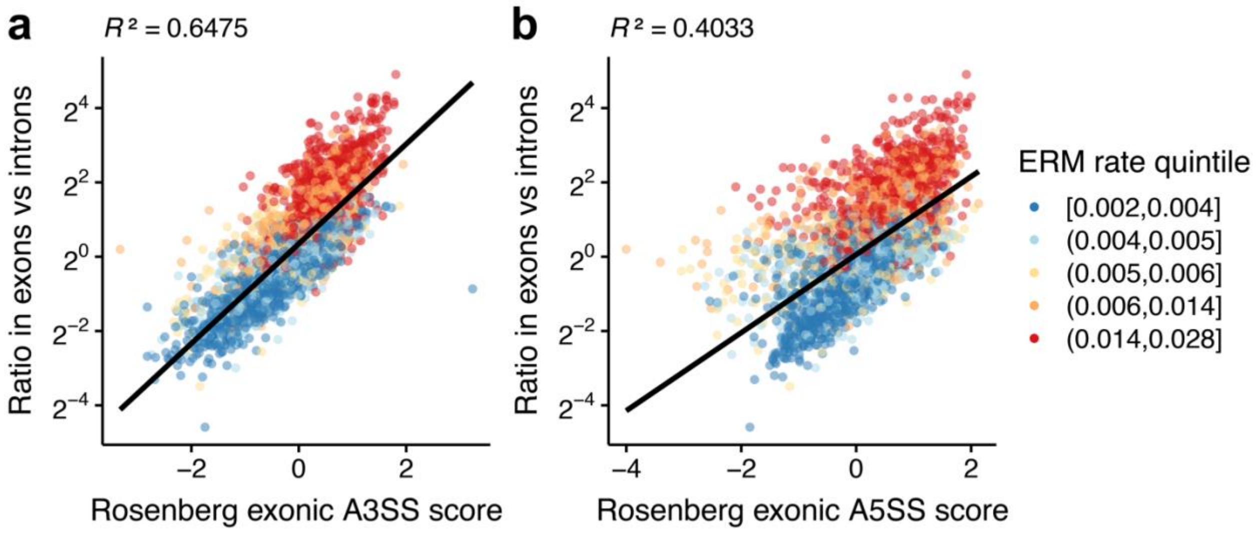

Supplementary Figure 2. Alternative measures of ESE activity do not affect results. Motifs enriched in exons versus introns (y-axis) exhibit higher ESE activity when using (x-axis), and exhibit higher mutability (ERM rate quintiles). Compared to Fig. 2d, two alternative measures of hexamer ESE activity (A3SS and A5SS) were used instead of EI scores: Rosenberg **(a)** exonic A3SS and **(b)** exonic A5SS score of ESE activity is based on the effect ($\log_2$ odds ratio) of exonic hexamers on alternative 3'/5' splice site usage as measured by high throughput minigene experiments[25]. **(a,b)** Squared Pearson's correlations (*n*=4,096 hexamers). Source data are provided as a Source Data file.

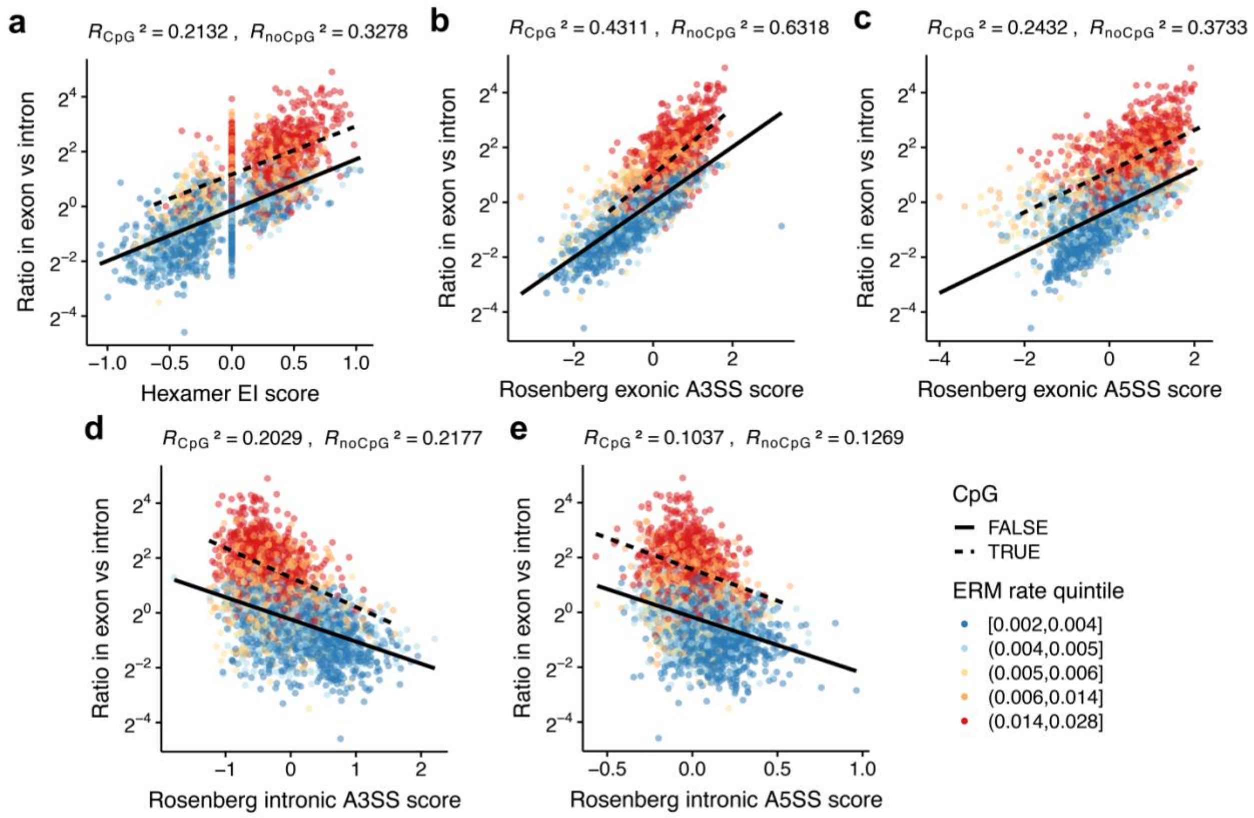

Supplementary Figure 3. CpG content of motifs does not affect results. Both motifs with (dashed lines, CpG) or without CpG dimers (solid lines, noCpG) are enriched in exons versus introns and exhibit higher/lower ESE/ISE activity. **(a)** As in Fig. 2d, ESE activity is measured by EI score. **(b-c)** As in Supplementary Fig. 2, ESE activity is measured by Rosenberg exonic A3SS and A5SS scores. **(d-e)** As in Fig. 5, ISE activity is measured by Rosenberg intronic A3SS and A5SS scores. Motifs are grouped by mutability (ERM rate quintiles). **(a-e)** Squared Pearson's correlations (*n*=1,185 hexamers with CpG, 2,911 hexamers without CpG). Source data are provided as a Source Data file.

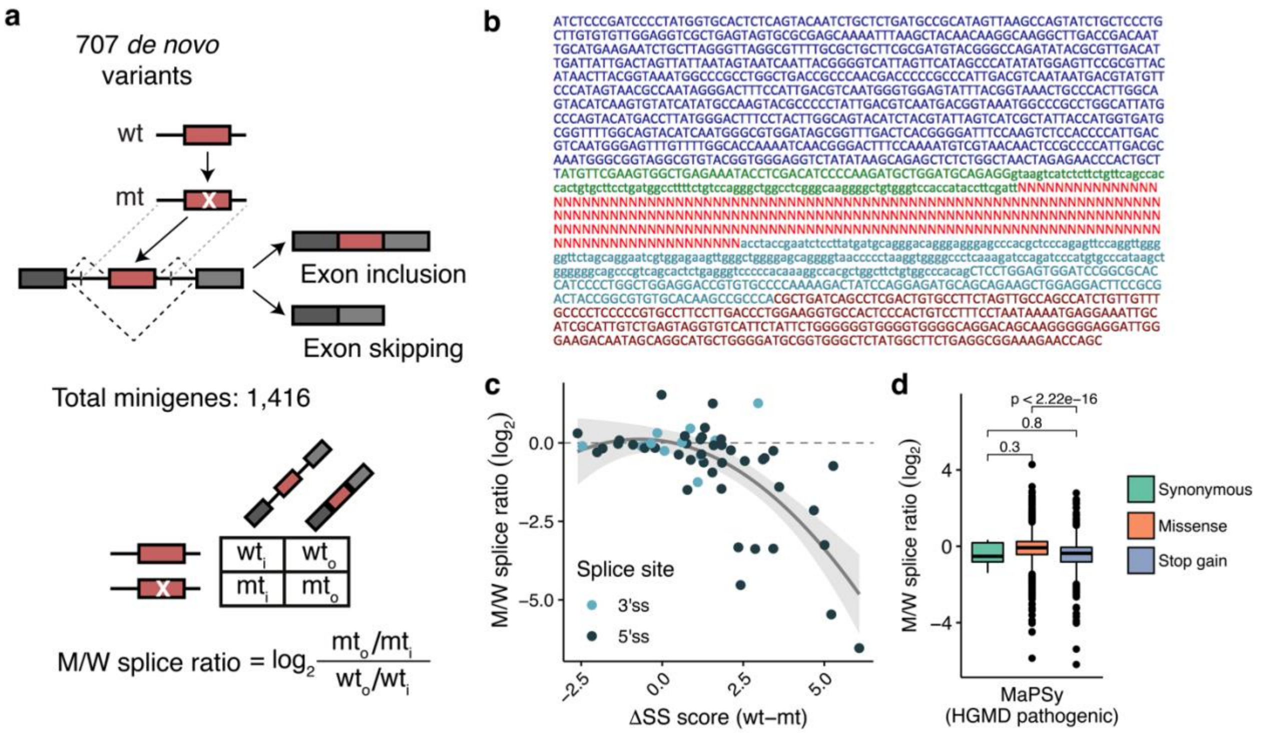

Supplementary Figure 4. High throughput splicing assay for 707 *de novo* mutations. (a) A panel of 707 Simons Simplex Collection *de novo* mutant and wild type pairs were synthesized and incorporated into a three-exon minigene library comprised of 1,414 minigenes. The pooled library was sequenced (input), transfected into HEK 293T tissue culture cells, and cDNA was generated and sequenced (output). The sequencing read counts in the input (wt_i, mt_i in the contingency table) and output (wt_o, mt_o) were used to calculate the mutant to wild type splice ratios (M/W splice ratio). **(b)** The *in vivo* splicing assay minigene reporter construct: CMV enhancer and promoter sequence (blue), exon 7 of ACTN4 (uppercase, green) and intron 7 of ACTN4 (lowercase, green), 230-mer library (red), intron 9 of ACTN4 (lowercase, cyan) and exon 10 of ACTN4 (uppercase, cyan), bGH poly(a) (maroon). **(c)** Comparison between the severity of 54 *de novo* mutations in the assay in either 3' (blue) and 5' splice site regions (black) as measured by their change in MaxEntScan score (ΔSS score (wt-mt)) or by their *in vivo MaPSy* splicing disruption results (M/W splice ratio). Quadratic polynomial regression (95% confidence band). **(d)** Comparison between the severity of splicing disruption (M/W splice ratio, $\log_2$) versus severity of protein disruption (synonymous < missense < stop gain) for previously published *in vitro* MaPSy results[29]. *In vitro* results are robust to nonsense-mediated decay (n=7 synonymous, 2,745 missense, 782 stop gain variants). The boxplots indicate the median (middle line), first and third quartiles (box), and 1.5x IQR (whiskers); pairwise *p*-values from Mann-Whitney two-tailed test. Source data are provided as a Source Data file.

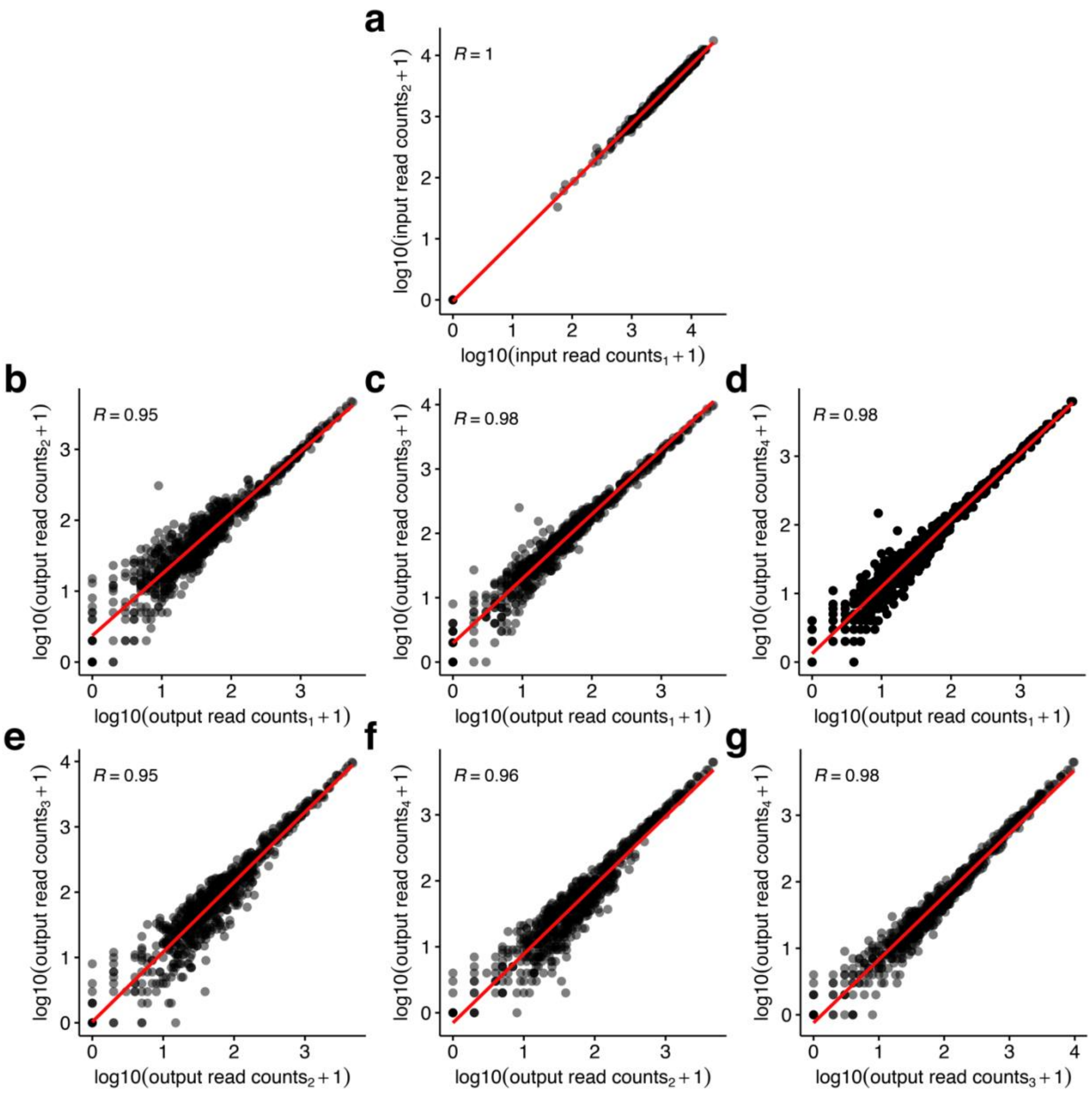

Supplementary Figure 5. Replication of input and output read counts in the splicing assay of *de novo* variants. Uniquely mapped read counts for both mutant and wildtype species were compared for **(a)** two technical sequencing replicates of the unspliced minigene reporter (input replicates), and **(b-g)** four replicate transfections followed by sequencing of the spliced cDNA (output replicates). **(a-g)** Pearson's correlations (*n*=1,414 mutant and wild type sequences). Source data are provided as a Source Data file.

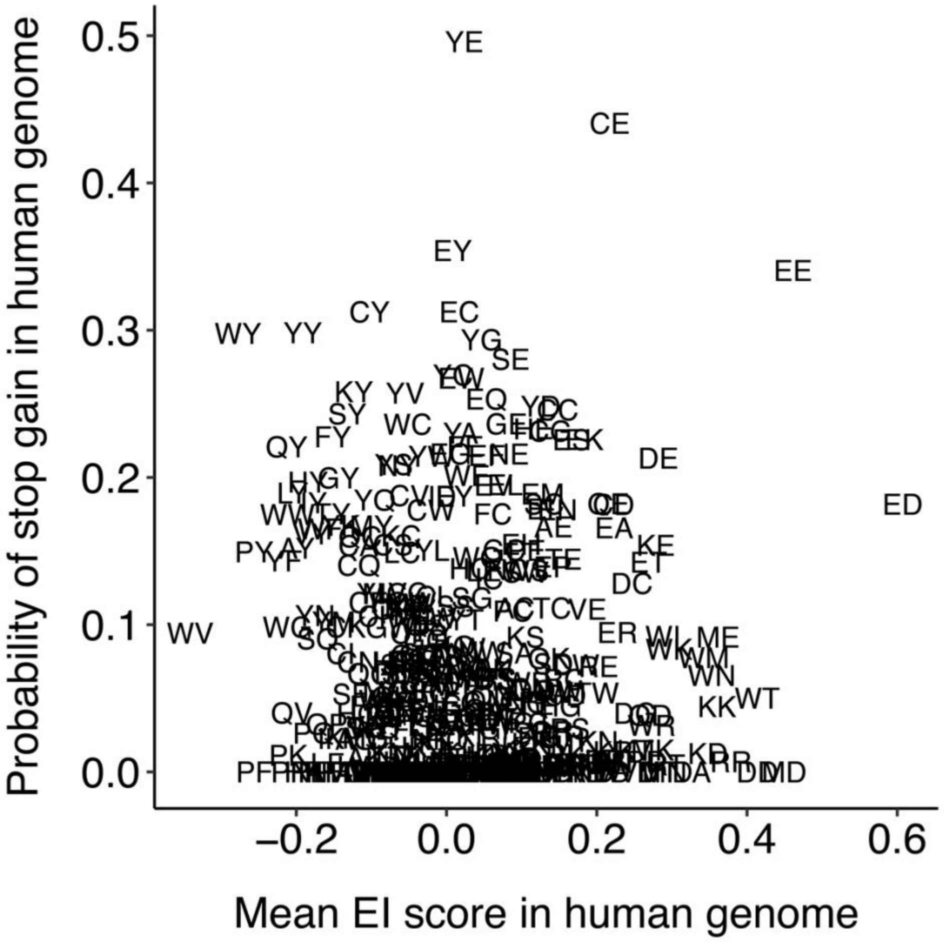

Supplementary Figure 6. EE amino acid pairs have among the highest probability of stop gain mutations. The probability of mutating to a stop gain was calculated for each amino acid pair by the count of all possible stop gain mutations overlapping instances of the amino acid pair in the human genome weighted by each mutation's mutability (ERM rate) divided by the count of all possible mutations of any type overlapping the amino acid pair weighted by mutability. EE ranks fourth behind YE, CE, and EY (out of 400 amino acid pairs). Source data are provided as a Source Data file.

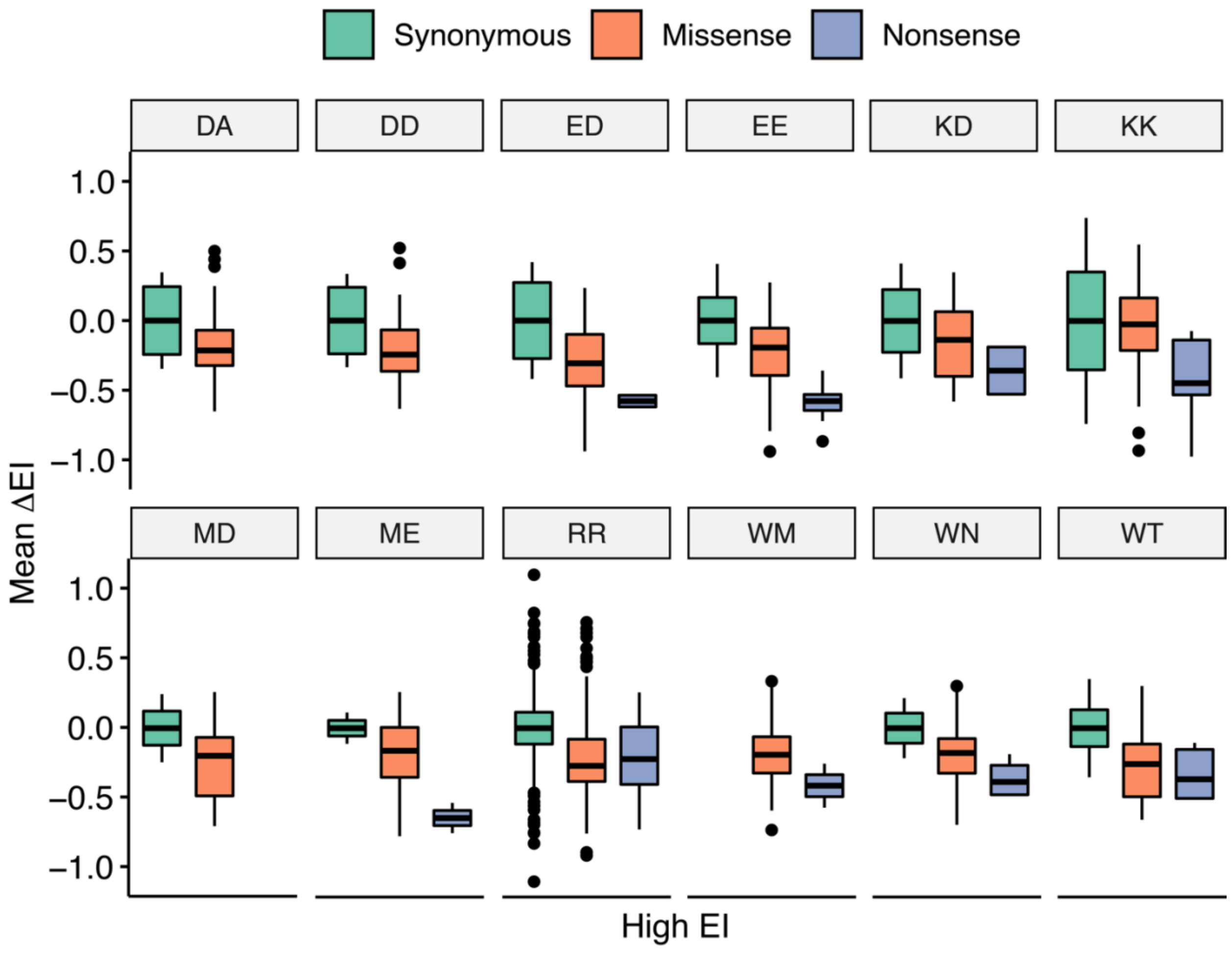

Supplementary Figure 7. Splice disruption is mostly concordant with protein disruption for amino acid pairs with high EI. As in Fig. 4, the change in exonic splicing enhancer activity (mean ΔEI) of possible mutations in the amino acid pairs with high EI (>0.3) (DA: *n*=144 mutations, DD: 72, ED: 72, EE: 72, KD: 72, KK: 72, MD: 36, ME: 36, RR: 648, WM: 18, WN: 36, WT: 72). Missense mutations were predicted to be more splicing disruptive on average than synonymous mutations, except for the case of WM (no synonymous mutations possible). Stop gain mutations were generally more splicing disruptive than missense mutations, except for the cases of DA, DD, and MD (no stop gains mutations possible), and RR (slightly less disruptive). Boxplots indicate the median (middle line), first and third quartiles (box), and 1.5x IQR (whiskers). Source data are provided as a Source Data file.

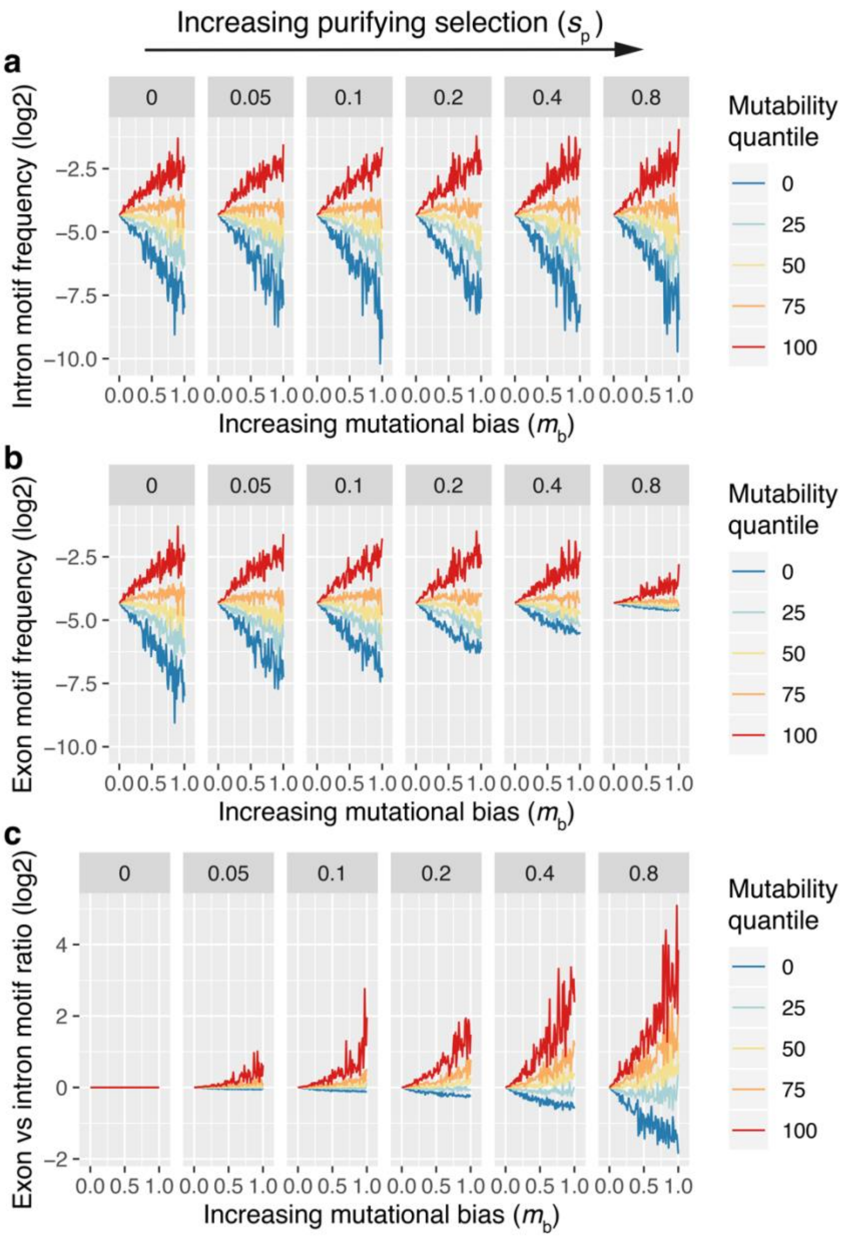

Supplementary Figure 8. Mutational bias and protein selection are both necessary to create pre-ESEs and pre-ISEs. A mathematical model of motif evolution under different levels of mutational bias (m_b parameter) and different levels of purifying selection in exons (s_p parameter) was developed, assuming non-overlapping loci (independently evolving), infinite population size and genome size (no genetic drift), and mutation-selection equilibrium (Supplementary Note 1). The mutation-selection equilibrium for **(a)** intron motif frequencies, **(b)** exon motif frequencies, and **(c)** exon vs intron motif ratios was solved using Supplementary Equations 1, 2, and 3, respectively, for randomly sampled 20x20 mutation rate matrices and different values of m_b and s_p (Supplementary Note 1). **(a,b)** Increasing m_b results in more variable motif frequencies in introns and exons, **(b)** but increasing s_p results in more constrained motif frequencies in exons. **(c)** Increasing both m_b and s_p results in motifs with more extreme enrichments in exons (pre-ESEs) and introns (pre-ISEs), depending on their relative mutability (Mutability quintile).

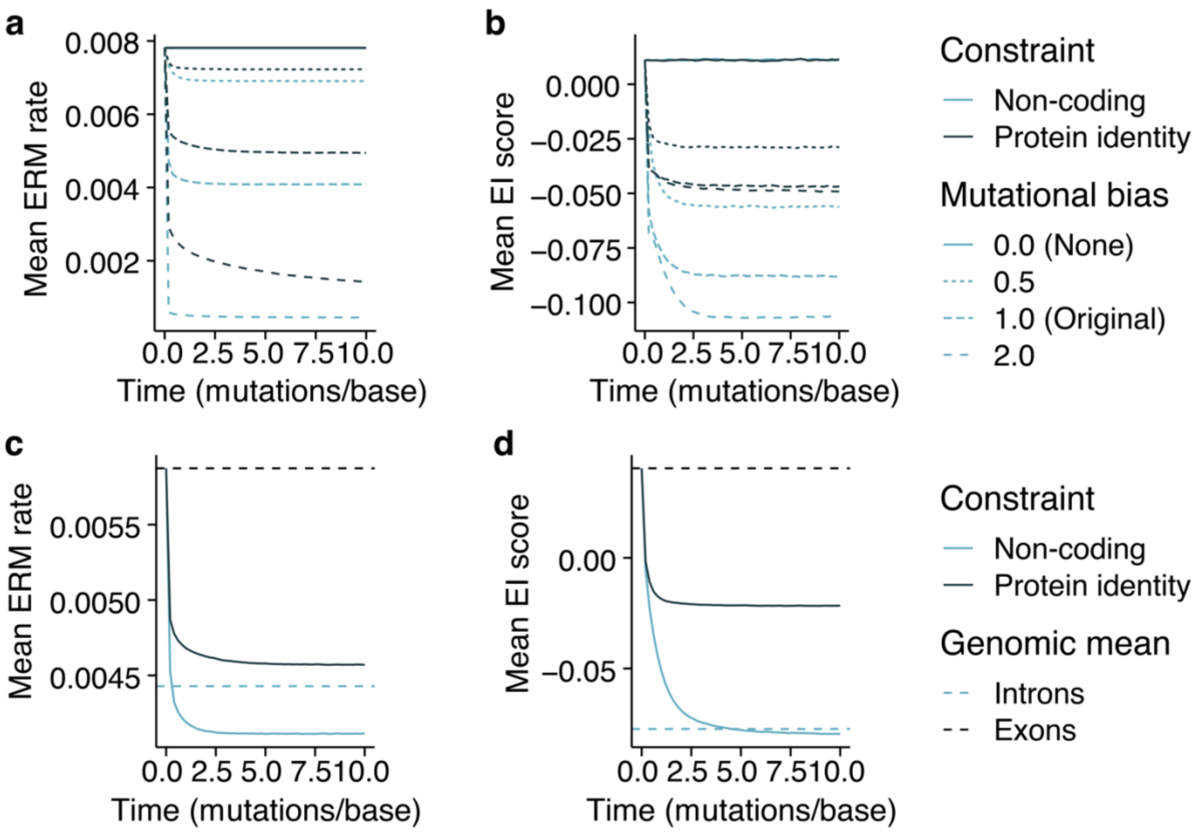

Supplementary Figure 9. Simulations with varying levels of mutational bias and an estimate of intrinsic ESE activity in the protein code. Simulations initialized on a genome of random sequence were run to model evolution with or without protein selection and with varying levels of mutational bias (Methods). **(a)** Mutability (mean ERM rate) and **(b)** ESE activity (mean EI score) were recorded over time (x-axis). For uniform mutation rates, mutability and ESE activity remain near initial levels. Increasing mutational bias resulted in greater decreases in mutability and ESE activity. Simulations with protein selection (black, analogous to exons) retained mutability and ESE activity at higher levels compared to simulations without selection (blue, analogous to introns). Simulations were also initialized on a genome of human exonic sequence, again recording **(c)** mutability and **(d)** ESE activity. Human values of exonic and intronic mutability and ESE activity (dotted lines) were compared against simulations (solid lines). Simulations without protein selection equilibrated at ESE levels similar to human intronic values (blue), whereas simulations with selection equilibrated at ESE levels about half the distance between human intronic and exonic values (black), suggesting about half the ESE activity in the human genome resides in the protein code.

Supplementary Note 1 for 'Mutational bias and the protein-code shape the evolution of splicing enhancers'

Rong et al.

A model with mutational bias and purifying selection in exons

We present a model of sequence evolution with mutational bias (motifs with variable mutation rates) and purifying selection in exons (to preserve protein-coding function) but not in introns. We show that the joint interaction of these two forces results in particular motifs becoming enriched in exons relative to introns at equilibrium, and vice versa.

Let G_{intron} and G_{exon} be the sets of intronic and exonic loci in a genome, respectively, where we assume G_{intron} and G_{exon} are disjoint and both consist of infinitely-many loci. Define a locus as a random variable whose state is one of n possible motifs. For example, in DNA, the set of possible motifs at a base position may be defined by the set of $n = 4^k$ possible k-mers, when considering a sequence context of length k bps centered at the base position (as in the Main Text, where $k = 7$). Let us further partition G_{exon} into the subset of loci evolving neutrally, $G_{\text{exon,ne}}$, and the subset of loci evolving under purifying selection, $G_{\text{exon,ps}}$, and assume G_{intron} consists of only neutrally evolving loci. For simplicity, assume loci are non-overlapping and independently evolving, such that their position relative to each other does not matter (loci are exchangeable within G_{intron}, $G_{\text{exon,ne}}$, and $G_{\text{exon,ps}}$). This assumption is relaxed in the simulations described in the Main Text, where we explicitly simulate mutations based on overlapping heptamer contexts along a linked sequence.

The assumptions of exchangeable and infinitely-many loci imply we only need to keep

track of the proportion of loci in G_{intron}, $G_{\text{exon,ne}}$, and $G_{\text{exon,ps}}$ in each of the n possible motif states over time. We want to model the evolution of motif proportions as a system of ordinary differential equations. Define the n length vector representation $\mathbf{p}_{\text{intron}}(t) = \{p_{\text{intron},i}(t)\}$ as the proportions of loci in G_{intron} found in each of the motif states indexed from $1, \cdots, n$ at time $t \geq 0$, where $\sum_{i=1}^{n} p_i(t) = 1$. Similarly, define $\mathbf{p}_{\text{exon,ne}}(t)$ with respect to $G_{\text{exon,ne}}$, and $\mathbf{p}_{\text{exon,ps}}(t)$ with respect to $G_{\text{exon,ps}}$. Finally, define $\mathbf{p}_{\text{exon}}(t)$ with respect to G_{exon}, and let s_{p} be the proportion of exonic loci evolving under purifying selection, such that

$$\mathbf{p}_{\text{exon}}(t) = (1 - s_{\text{p}})\mathbf{p}_{\text{exon,ne}}(t) + s_{\text{p}}\mathbf{p}_{\text{exon,ps}}(t).$$

Define a mutation rate matrix for n motifs as

$$\mathbf{M} = \begin{bmatrix} -\sum_{k\neq 1} m_{1,k} & m_{1,2} & \cdots & m_{1,n} \\ m_{2,1} & -\sum_{k\neq 2} m_{2,k} & \cdots & m_{2,n} \\ \vdots & \vdots & \ddots & \vdots \\ m_{n,1} & m_{n,2} & \cdots & -\sum_{k\neq i} m_{n,k} \end{bmatrix},$$

where the off-diagonals $m_{i,j} \geq 0$ represent the rates at which motif i mutates to motif j, the diagonals $-\sum_{k\neq i} m_{i.k}$ represent the rates at which motif i mutates, and assume $\mathbf{M}$ is invertible. For a diploid population with N individuals, define the substitution rate matrix for n motifs as

$$\mathbf{M}^* = 2NU\mathbf{M},$$

where $2N\mathbf{M}$ is the population-level mutation rate matrix, and U is the probability of fixation of a mutant motif. Under neutral evolution, we have $U = 1/(2N)$, thus

$$\mathbf{M}^*_{\text{intron}} = \mathbf{M}^*_{\text{exon,ne}} = \mathbf{M}.$$

To model purifying selection, assume a selective coefficient $s < 0$. The probability of

fixation of a new mutation is $U = (1 - e^{-2s})/(1 - e^{-4Ns})$ according to Kimura[66]. Thus,

$$\mathbf{M}^*_{\text{exon,ps}} = 2N\frac{1 - e^{-2s}}{1 - e^{-4Ns}}\mathbf{M},$$

and $\mathbf{M}^*_{\text{exon}} = (1 - s_{\text{p}})\mathbf{M}^*_{\text{exon,ne}} + s_{\text{p}}\mathbf{M}^*_{\text{exon,ps}} = (1 - s_{\text{p}})\mathbf{M} + s_{\text{p}}\mathbf{M}^*_{\text{exon,ps}}$. If we assume either infinite population size ($N \to \infty$), or extremely strong purifying selection ($s \to -\infty$), we have $U \to 0$, thus $\mathbf{M}^*_{\text{exon,ps}} = 0_{n,n}$, and $\mathbf{M}^*_{\text{exon}} = (1 - s_{\text{p}})\mathbf{M}$.

Thus, the evolution of exonic and intronic motif proportions can be modeled by the following system of ordinary differential equations:

$$\begin{aligned}
\frac{d}{dx}\mathbf{p}_{\text{intron}}(t) &= \mathbf{p}(t)\mathbf{M}^*_{\text{intron}}, \\
\frac{d}{dx}\mathbf{p}_{\text{exon,ne}}(t) &= \mathbf{p}(t)\mathbf{M}^*_{\text{exon,ne}}, \\
\frac{d}{dx}\mathbf{p}_{\text{exon,ps}}(t) &= \mathbf{p}(t)\mathbf{M}^*_{\text{exon,ps}}, \\
\frac{d}{dx}\mathbf{p}_{\text{exon}}(t) &= (1 - s_{\text{p}})\mathbf{p}(t)\mathbf{M}^*_{\text{exon,ne}} + s_{\text{p}}\mathbf{p}(t)\mathbf{M}^*_{\text{exon,ps}}.
\end{aligned}$$

Define the mutation-selection-drift equilibrium of $\mathbf{p}_{\text{intron}}(t)$ as $\boldsymbol{\pi}_{\text{intron}}$, which satisfies

$$\boldsymbol{\pi}_{\text{intron}}\mathbf{M}^*_{\text{intron}} = \boldsymbol{\pi}_{\text{intron}}\mathbf{M} = 0_{n,n}, \text{ and } \sum_{i=1}^{n} \pi_{\text{intron},i} = 1.$$

We can solve this system by defining augmented matrices

$$\mathbf{M}_\alpha = \begin{bmatrix} -\sum_{k\neq 1} m_{1,k} & m_{1,2} & \cdots & m_{1,n-1} & 1 \\ m_{2,1} & -\sum_{k\neq 2} m_{2,k} & \cdots & m_{2,n-1} & 1 \\ \vdots & \vdots & \ddots & \vdots & \vdots \\ m_{n-1,1} & m_{n-1,2} & \cdots & -\sum_{k\neq n-1} m_{n-1,k} & 1 \\ m_{n,1} & m_{n,2} & \cdots & m_{n,n-1} & 1 \end{bmatrix},$$

and

$$\mathbf{e}_\alpha = [0, 0, \cdots, 0, 1].$$

This gives us the equivalent system

$$\boldsymbol{\pi}_{\text{intron}} \mathbf{M}_\alpha = \mathbf{e}_\alpha,$$

which has the solution

$$\boxed{\boldsymbol{\pi}_{\text{intron}} = \mathbf{e}_\alpha \mathbf{M}_\alpha^{-1}}. \tag{1}$$

Define the mutation-selection-drift equilibrium of $\mathbf{p}_{\text{exon}}(t)$ as $\boldsymbol{\pi}_{\text{exon}}$, of $\mathbf{p}_{\text{exon,ne}}(t)$ as $\boldsymbol{\pi}_{\text{exon,ne}}$, and of $\mathbf{p}_{\text{exon,ps}}(t)$ as $\boldsymbol{\pi}_{\text{exon,ps}}$. Since $\mathbf{M}^*_{\text{exon,ne}} = \mathbf{M}$, we have

$$\boldsymbol{\pi}_{\text{exon,ne}} = \mathbf{e}_\alpha \mathbf{M}_\alpha^{-1}.$$

Moreover, $\boldsymbol{\pi}_{\text{exon,ps}}$ by definition satisfies

$$\boldsymbol{\pi}_{\text{exon,ps}} \mathbf{M}^*_{\text{exon}} = 2N \frac{1 - e^{-2s}}{1 - e^{-4Ns}} \boldsymbol{\pi}_{\text{exon,ps}} \mathbf{M} = 0, \text{and } \sum_{i=1}^{n} \pi_{\text{exon,ps},i} = 1.$$

We can solve this system by similarly defining the augmented matrix

$$\mathbf{M}^*_\alpha = 2N \frac{1 - e^{-2s}}{1 - e^{-4Ns}} \begin{bmatrix} -\sum_{k \neq 1} m_{1,k} & m_{1,2} & \cdots & m_{1,n-1} & 1 \\ m_{2,1} & -\sum_{k \neq 2} m_{2,k} & \cdots & m_{2,n-1} & 1 \\ \vdots & \vdots & \ddots & \vdots & \vdots \\ m_{n-1,1} & m_{n-1,2} & \cdots & -\sum_{k \neq n-1} m_{n-1,k} & 1 \\ m_{n,1} & m_{n,2} & \cdots & m_{n,n-1} & 1 \end{bmatrix}.$$

This gives us the equivalent system

$$\boldsymbol{\pi}_{\text{exon,ps}} \mathbf{M}^*_\alpha = \mathbf{e}_\alpha,$$

which has the solution

$$\boldsymbol{\pi}_{\text{exon,ps}} = \begin{cases} \mathbf{p}_{\text{exon,ps}}(0), & \text{if } \mathbf{M}^* = 0_{n,n} \\ \mathbf{e}_\alpha (\mathbf{M}^*_\alpha)^{-1}, & \text{otherwise} \end{cases}.$$

Thus,

$$\boxed{\boldsymbol{\pi}_{\text{exon}} = \begin{cases} (1-s_{\text{p}})\mathbf{e}_\alpha \mathbf{M}_\alpha^{-1} + s_{\text{p}}\mathbf{p}_{\text{exon,ps}}(0), & \text{if } \mathbf{M}^* = 0_{n,n} \\ (1-s_{\text{p}})\mathbf{e}_\alpha \mathbf{M}_\alpha^{-1} + s_{\text{p}}\mathbf{e}_\alpha(\mathbf{M}^*_\alpha)^{-1}, & \text{otherwise} \end{cases}}. \tag{2}$$

Finally, we want the ratios of motif proportions in exons relative to introns at mutation-selection-drift equilibrium (division is element-wise):

$$\boxed{\mathbf{r}_{\text{equil}} = \frac{\boldsymbol{\pi}_{\text{exon}}}{\boldsymbol{\pi}_{\text{intron}}} = \begin{cases} (1-s_{\text{p}})\mathbf{1}_n + \dfrac{s_{\text{p}}\mathbf{p}_{\text{exon}}(0)}{\mathbf{e}_\alpha}\mathbf{M}_\alpha^{-1}, & \text{if } \mathbf{M}^* = 0_{n,n} \\ (1-s_{\text{p}})\mathbf{1}_n + \dfrac{s_{\text{p}}\mathbf{e}_\alpha(\mathbf{M}^*_\alpha)^{-1}}{\mathbf{e}_\alpha\mathbf{M}_\alpha^{-1}}, & \text{otherwise} \end{cases}}. \tag{3}$$

Note that the $\mathbf{M}^* = 0_{n,n}$ case corresponds to the mutation-selection equilibrium that arises when the probability of fixation of motifs under purifying selection by genetic drift is zero. (Recall that $\mathbf{M}^* = 0_{n,n}$ if we assume either infinite population size or extremely strong purifying selection.) Ignoring genetic drift, the ratios at mutation-selection equilibrium, $\mathbf{r}_{\text{equil}}$, depend on the initial exonic motif proportions, $\mathbf{p}_{\text{exon}}(0)$, the proportion of loci under purifying selection, s_{p}, and the specific mutation rate matrix, $\mathbf{M}$. If no exonic loci are under purifying selection ($s_{\text{p}} = 0$), then $\mathbf{r}_{\text{equil}} = \mathbf{1}_n$. If loci in exons are under extremely weak purifying selection ($s \to 0$), then $U \to 1/(2N)$, $\mathbf{M}^*_{\text{exon,ps}} = \mathbf{M}$, and $\mathbf{r}_{\text{equil}} = \mathbf{1}_n$. If $\mathbf{M}$ has no mutational bias, that is, all off-diagonals are equal, then $\boldsymbol{\pi}_{\text{intron}} = \mathbf{1}_n/n$. If we make the additional assumption that $\mathbf{p}_{\text{exon}}(0)$ is approximately uniform to begin with ($\mathbf{p}_{\text{exon}}(0) \approx \mathbf{1}_n/n$), then $\mathbf{r}_{\text{equil}} \approx \mathbf{1}_n$. Under these assumptions, both purifying selection ($s_{\text{p}} > 0$ or $s < 0$) and mutational bias ($\mathbf{M}$ with non-equal off-diagonal entries) are necessary to enrich for motifs in exons relative to introns.

In Supplementary Fig. 8, we created a family of mutation rate matrices of size n motifs $\{\mathbf{M}(m_\mathrm{b})\}$ with varying levels of a mutational bias parameter m_b (see below), where $\mathbf{M}(m_\mathrm{b})$ is sampled as follows: randomly sample $m_{ij} = e^{x_i} e^{y_{ij}}$, where $x_i \sim \mathrm{Normal}(0, m_\mathrm{b})$ for each $1 \leq i \leq n$, and $y_{ij} \sim \mathrm{Normal}(0, m_\mathrm{b})$ for each $1 \leq j \leq n$ and $j \neq i$. Here, e^{x_i} represents a base-line mutation rate for motif i, and $e^{y_{ij}}$ represents a modifier of the base-line mutation rate for the mutation rate of each motif i to each motif j. If $m_\mathrm{b} = 0$, then $\mathbf{M}(m_\mathrm{b})$ has no mutational bias. As we increase m_b, $\mathbf{M}$ gains larger and smaller off-diagonal entries, consistent with increasing mutational bias. In Supplementary Fig. 8, we plotted quantiles of **(a)** $\boldsymbol{\pi}_\mathrm{intron}$, **(b)** $\boldsymbol{\pi}_\mathrm{exon}$, and **(c)** $\mathbf{r}_\mathrm{equil}$ using Supplementary Equations 1, 2, 3, respectively. We assumed no genetic drift and uniform initial exonic proportions for $n = 20$ motifs, let $s_\mathrm{p} \in \{0, 0.05, 0.1, 0.2, 0.4, 0.8\}$, and randomly generated $\mathbf{M}(m_\mathrm{b})$ for each $m_\mathrm{b} \in \{0, 0.01, 0.02, 0.03, \cdots, 1\}$. We show that as we increase (or decrease) s_p and m_b, the spread of exon versus intron motif ratios also increases (or decreases). In the main text, we argue this drives ESE and ISE evolution.
