# Supplementary material for: Mutational bias and the protein code shape the evolution of splicing enhancers
Source: Nat Commun. 2020 Jun 5;11:2845. doi: 10.1038/s41467-020-16673-z (PMC7275064; doi:10.1038/s41467-020-16673-z)
Supplement: Supplementary file 2 — Reporting Summary [file 41467_2020_16673_MOESM2_ESM.pdf]

nature research

Corresponding author(s): William G. Fairbrother (PhD)

Last updated by author(s): Mar 30, 2020

Reporting Summary

Nature Research wishes to improve the reproducibility of the work that we publish. This form provides structure for consistency and transparency in reporting. For further information on Nature Research policies, see Authors & Referees and the Editorial Policy Checklist .

Statistics

For all statistical analyses, confirm that the following items are present in the figure legend, table legend, main text, or Methods section.

n/a	Confirmed	
☐	☒	The exact sample size (*n*) for each experimental group/condition, given as a discrete number and unit of measurement
☐	☒	A statement on whether measurements were taken from distinct samples or whether the same sample was measured repeatedly
☐	☒	The statistical test(s) used AND whether they are one- or two-sided<br>*Only common tests should be described solely by name; describe more complex techniques in the Methods section.*
☐	☒	A description of all covariates tested
☐	☒	A description of any assumptions or corrections, such as tests of normality and adjustment for multiple comparisons
☐	☒	A full description of the statistical parameters including central tendency (e.g. means) or other basic estimates (e.g. regression coefficient) AND variation (e.g. standard deviation) or associated estimates of uncertainty (e.g. confidence intervals)
☐	☒	For null hypothesis testing, the test statistic (e.g. *F*, *t*, *r*) with confidence intervals, effect sizes, degrees of freedom and *P* value noted<br>*Give P values as exact values whenever suitable.*
☒	☐	For Bayesian analysis, information on the choice of priors and Markov chain Monte Carlo settings
☒	☐	For hierarchical and complex designs, identification of the appropriate level for tests and full reporting of outcomes
☐	☒	Estimates of effect sizes (e.g. Cohen's *d*, Pearson's *r*), indicating how they were calculated

Our web collection on statistics for biologists contains articles on many of the points above.

Software and code

Policy information about availability of computer code

Data collection	No software was used for data collection.
Data analysis	R software (3.4.4) was used for statistical analysis in all figures.<br><br>SnpEff (4.3T) was use to annotate variants for functional effects.<br><br>STAR aligner (2.5.1b) was used to align sequencing reads in the splicing assay of de novo variants.<br><br>We developed custom scripts for the evolutionary simulations and the mathematical model (https://github.com/stephenrong/mutation-paper, DOI: 10.5281/zenodo.3727198, MIT License).

For manuscripts utilizing custom algorithms or software that are central to the research but not yet described in published literature, software must be made available to editors/reviewers. We strongly encourage code deposition in a community repository (e.g. GitHub). See the Nature Research guidelines for submitting code & software for further information.

Data

Policy information about availability of data

All manuscripts must include a data availability statement. This statement should provide the following information, where applicable:
- Accession codes, unique identifiers, or web links for publicly available datasets
- A list of figures that have associated raw data
- A description of any restrictions on data availability

The authors declare the data supporting the findings of this study are available within the paper and its supplementary files. The source data underlying Figs 2c,d, 3a–d, 4a–e, 5a,b, Supplementary Figs 1, 2a,b, 3a–e, 4c–d, 5a–g, 6, and 7 are provided as a Source Data File. Other datasets referenced in this study are available from the following web links: ERM rates [https://www.nature.com/articles/s41467-018-05936-5#Sec22], EI scores [https://genome.cshlp.org/content/21/8/1360/

suppl/DC1], Rosenberg intronic and exonic A3SS and A5SS scores [https://github.com/Alex-Rosenberg/cell-2015/blob/master/ipython.notebooks/Cell2015_N4_Motif_Effect_Sizes.ipynb], ExAC variant annotations [https://github.com/macarthur-lab/exac_2015], MaxEntScan scores [http://hollywood.mit.edu/burgelab/maxent/Xmaxentscan_scoreseq.html], MaPSy in vivo and in vivo splicing assay results for HGMD pathogenic variants [http://fairbrother.biomed.brown.edu/data/bulk_download.txt], Vex-seq splicing assay results [https://github.com/scottiadamson/Vex-seq/blob/master/processed_files/delta_PSI_values.tsv], and MFASS splicing assay results [https://github.com/KosuriLab/MFASS/blob/master/processed_data/snv/snv_data_clean.txt].

Field-specific reporting

Please select the one below that is the best fit for your research. If you are not sure, read the appropriate sections before making your selection.

☒ Life sciences ☐ Behavioural & social sciences ☐ Ecological, evolutionary & environmental sciences

For a reference copy of the document with all sections, see nature.com/documents/nr-reporting-summary-flat.pdf

Life sciences study design

All studies must disclose on these points even when the disclosure is negative.

Sample size

707 de novo mutations were selected for analysis because this was the number of variants in Simons Simplex Collection that were available to be analyzed in the splicing assay given exon size constraints of the minigene construct (i.e. <= 115). We also reanalyzed data from three high throughput splicing studies of human genetic variants. Our reanalysis of Soemedi et al. (2017) had a sample size of 3,707 variants from HGMD, limited to exons of length <= 110 nt by the constraints of the MaPSy assay. Our reanalysis of Adamson et al. (2018) had a sample size of 782 ExAC variants, limited to 110 randomly chosen exons of length <= 97 nt by constraints of the Vex-seq assay. Our reanalysis of Cheung et al. (2019) had a sample size of 12,783 ExAC variants, limited to 2,920 exons of length <= 100 nt by constraints of the MFASS assay. These four assays constitute all four published high throughput splicing assays of human genetic variants at genomic scale.

For analysis of hexamer mutation rates and splicing scores, we analyzed all 4,096 possible hexamers motifs. For analysis of amino acid pairs, we analyzed all 400 possible amino acid pairs. For the analysis of ExAC variants, we used 889K exonic variants with allele frequencies > 5e-5.

Data exclusions

For the splicing assay of de novo variants in the Simons Simplex Collection, we synthesized all 707 exonic de novo variants that mapped to human exons <= 115 nt in length. Since 230 nt is the current limit for sequence length in high throughput oligonucleotide synthesis, we were limited to analyzing variants in exons that were substantially shorter than 230 nt. A cutoff of 115 nt for exon length was chosen to allow for synthesis of the full exon sequence, 15 nt of the downstream intron, at least 50 nt of the upstream intron, and 25 nt primers on both ends of the sequence. The 15 nt of downstream intron and 50 nt of upstream intron were chosen so that the oligonucleotide includes both the 3' and 5' splice sites and the upstream branchpoint sequence (generally within 50 nt) required for proper splicing. Only exonic variants were analyzed because the downstream analysis of spliced sequences can uniquely distinguish exonic, but not intronic, variants.

In analyzing the de novo splicing assay results, we wanted to compare the effect of synonymous, missense, and stop gain variants on splicing. We thus removed variants that were also annotated as splice region structural interaction, 5’ UTR, 3’ UTR, and sequence feature variants, since these additional variant effects may confound results.

In the analysis of ExAC variants, we removed variants with derived allele frequencies < 5e-5, which corresponds to variants with three or less allele counts. These rarest allele frequencies bins are the most strongly affected by recurrent mutation (Harpak et al. 2016). Inclusion of these variants would complicate analysis.

Replication

The biochemical assays were performed with two input minigene sequencing replicates and four independent transfections that were followed by sequencing of cDNA output. All four attempts at transfections were successful.

Randomization

Randomization is not relevant to this study. All 707 de novo mutations were analyzed in every input (2 replicates) and output library (4 replicates). The six input + output libraries were multiplexed and sequenced on a single Illumina HiSeq 3000 (2x250) lane.

Blinding

Blinding is not applicable because no scoring of experimental sets with perturbation/no perturbation was performed.

Reporting for specific materials, systems and methods

We require information from authors about some types of materials, experimental systems and methods used in many studies. Here, indicate whether each material, system or method listed is relevant to your study. If you are not sure if a list item applies to your research, read the appropriate section before selecting a response.

Materials & experimental systems

n/a	Involved in the study	
☒	☐	Antibodies
☐	☒	Eukaryotic cell lines
☒	☐	Palaeontology
☒	☐	Animals and other organisms
☒	☐	Human research participants
☒	☐	Clinical data

Methods

n/a	Involved in the study	
☒	☐	ChIP-seq
☒	☐	Flow cytometry
☒	☐	MRI-based neuroimaging

Eukaryotic cell lines

Policy information about cell lines

Cell line source(s)	HEK 293T cell line was obtained directly from ATCC (ATCC# CRL-3216).
Authentication	HEK 293T cell lines were not authenticated.
Mycoplasma contamination	HEK 293T cell line was confirmed mycoplasma free in previous passage.
Commonly misidentified lines (See ICLAC register)	HEK 293T cell line is not listed in the ICLAC Register of Misidentified Cell Lines (v10).
