# Supplementary material for: Mutational bias and the protein code shape the evolution of splicing enhancers
Source: Nat Commun. 2020 Jun 5;11:2845. doi: 10.1038/s41467-020-16673-z (PMC7275064; doi:10.1038/s41467-020-16673-z)
Supplement: Supplementary file 3 — Description of Additional Supplementary Files [file 41467_2020_16673_MOESM3_ESM.docx]

Description of Additional Supplementary Files

**File Name:** Supplementary Data 1

**Description:** De novo mutation splicing assay. Information on variants, exons, windows, aggregate read counts, M/W splice ratios, p-values, and FDR adjusted p-values.
